# PARP1 rewires neuroinflammatory and redox metabolism associated with reactive neuroglia in neuropathic pain

**DOI:** 10.1016/j.redox.2026.104268

**Published:** 2026-06-18

**Authors:** Simona Denaro, Simona D'Aprile, Anna Gervasi, Vincenzo Russo, Francesco Bellia, Sebastiano Giallongo, Alessandro Lavoro, Saverio Candido, Alice Braga, Alexander V. Gourine, Giovanni Li Volti, Lorella Pasquinucci, Angela Maria Amorini, Carmela Parenti, Rosalba Parenti, Nunzio Vicario

**Affiliations:** a Section of Physiology, Department of Biomedical and Biotechnological Sciences, University of Catania, Catania, 95123, Italy; b Department of Medicine and Surgery, University of Enna “Kore”, Enna, 94100, Italy; c Section of Biochemistry, Department of Biomedical and Biotechnological Sciences, University of Catania, Catania, 95123, Italy; d Section of General Pathology, Department of Biomedical and Biotechnological Sciences, University of Catania, Catania, 95123, Italy; e Centre for Cardiovascular and Metabolic Neuroscience, Neuroscience, Physiology and Pharmacology, University College London, London, WC1E 6BT, United Kingdom; f Section of Medicinal Chemistry, Department of Drug and Health Sciences, University of Catania, Catania, 95123, Italy; g Section of Pharmacology and Toxicology, Department of Drug and Health Sciences, University of Catania, Catania, 95123, Italy

**Keywords:** Reactive gliosis, Central sensitization, PAR polymers, Olaparib, Glutathione

## Abstract

Neuroinflammation, oxidative stress and metabolic dysfunction are co-dependent drivers of neuropathic pain, jointly sustaining reactive gliosis and spinal sensitization. The interplay among neuroinflammation, redox imbalance, and metabolic reprogramming in glial cells represents a crucial process in this context, but the integrative mechanisms involved remain elusive. Poly(ADP-ribose) polymerase 1 (PARP1), a nuclear enzyme activated by genotoxic stress, has emerged as a key regulator of neuroinflammatory-associated diseases. The aim of the present study was to investigate the role of PARP1 in the pathophysiology of neuropathic pain and to evaluate the neuroglia phenotype and metabolism. Using a combination of *in vitro* glial cell cultures and an *in vivo* model of peripheral nerve injury, we demonstrated that sustained PARP1 activation triggers parthanatos, apoptosis signalling, and reactive gliosis, promoting neuroinflammation and maladaptive redox-inflammatory coupling in the spinal cord. At the behavioural level, PARP1 inhibition attenuated mechanical allodynia and improved motor coordination in rats with chronic constriction injury (CCI) of the sciatic nerve. Multi-omics profiling revealed a broad restoration of injury-altered proteomic and metabolic signatures, converging on restoring redox imbalance and related metabolic pathways, including glutathione and amino acid metabolic processing. Cell-type-specific knockdown of PARP1 established a causal link between redox balance, mitochondrial metabolism, and intercellular crosstalk during microglia inflammatory priming. Taken together, these findings highlight PARP1 as a functional regulator of the glial cell reactive phenotype in neuropathic pain and support its inhibition as a strategy to modulate central sensitization mechanisms.

## Highlights

• A conserved Parp1-driven redox-metabolic signatures underlies neuropathic pain.
• Parp1 inhibition reduces allodynia, spinal gliosis and DNA damage.
• Multi-omics approaches reveal Parp1-dependent redox and metabolic rewiring.
• Parp1 emerges as a promising non-opioid target for neuropathic pain.

## Graphical abstract

**Figure:**
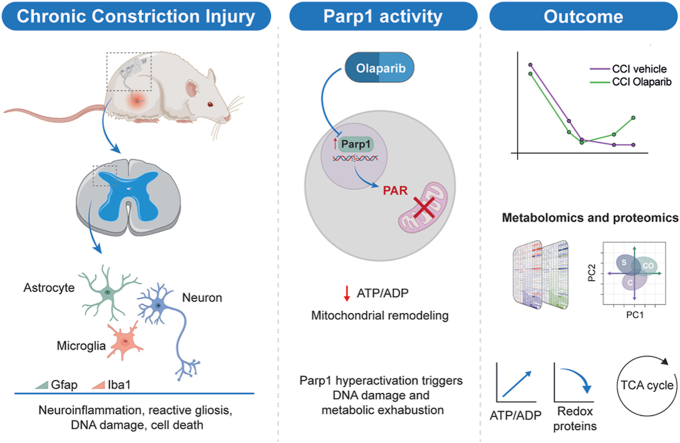

## 1 Introduction

Neuropathic pain is a multifaceted and chronic condition that affects millions of people, posing a significant public health challenge because of its debilitating consequences and impact on quality of life [1,2].

Currently, the available pain management therapies are insufficient to offer complete pain relief. Opioids are often prescribed for moderate to severe pain, with the current estimation indicating an average of nearly one opioid prescription per American [3,4]. However, the long-term use of opioids is associated with considerable risks, with overdose becoming a leading cause of death [5]. Consequently, there is an imperative demand for the exploitation of novel therapeutic targets to facilitate the development of cutting-edge treatment approaches. Mechanistically, neuropathic pain involves maladaptive neural plasticity accompanied by pathological neuroimmune interactions. A key contributor to this process is neuroinflammation, which is sustained by the interplay between the immune system and the nervous system and further disrupts synaptic excitability, promoting a persistent state of pain [6,7]. Additionally, oxidative stress and related metabolic dysfunction are recognized as key etiological factors that further aggravate pain pathology. Excessive production of reactive oxygen species (ROS) and impaired antioxidant defences induce lipid peroxidation, mitochondrial dysfunction, and DNA damage, ultimately leading to degeneration and cell death [[8], [9], [10]].

Poly(ADP-ribose) polymerase 1 (PARP1) is a key sensor of DNA strand breaks that orchestrates the cellular response to genotoxic stress in a NAD^+^-dependent manner [11,12]. Widely studied as a therapeutic target, PARP1 has been characterized for its role in DNA repair in the field of oncology [13,14]. However, sustained oxidative stress can lead to PARP1 overactivation, which causes the accumulation of poly(ADP-ribose) (PAR) polymers and rapid depletion of NAD^+^ and ATP, leading to a specific form of cell death marked by extensive DNA fragmentation, known as *parthanatos* [15]. In addition to its cytotoxic effect, PARP1 hyperactivation promotes inflammation by acting as a transcriptional coactivator of nuclear factor kappa B (NF-κB), thereby increasing the expression of proinflammatory genes and sustaining chronic central nervous system (CNS) inflammation [16]. These mechanisms have been recently recognized as significant contributors to neurodegenerative and neuroinflammatory diseases [17,18]. Several studies have reported that in Alzheimer disease and Parkinson disease PARP1 overactivation contributes to the accumulation of misfolded proteins and neuronal loss, as well as in ischaemia-reperfusion injury, where it exacerbates mitochondrial dysfunction and cell death [[19], [20], [21]]. However, despite its increasingly recognized role in neurodegeneration, the mechanistic contribution of PARP1 hyperactivation to the pathogenesis of neuropathic pain remains poorly understood.

Here, we investigated the contribution of PARP1 to neuropathic pain in a rat model of chronic constriction injury (CCI). Through integrated approaches, we characterized PARP1-dependent molecular alterations and assessed the effects of its inhibition with olaparib, a clinically approved PARP inhibitor used in cancer treatment. We further performed cell-type-specific PARP1 knockdown *in vitro* to support causal inference regarding PARP1 function and to assess its glial-autonomous effects.

## 2 Results

**PARP1 inhibition reduces the glial inflammatory response and mitochondrial dysfunction *in vitro***. Given the central role of glial cells in orchestrating the inflammatory response within the CNS [22,23], we established an *in vitro* co-culture model to test the impact of PARP1 hyperactivation on neuroglia and the efficacy of PARP1 inhibition via olaparib.

**Fig. 1:**
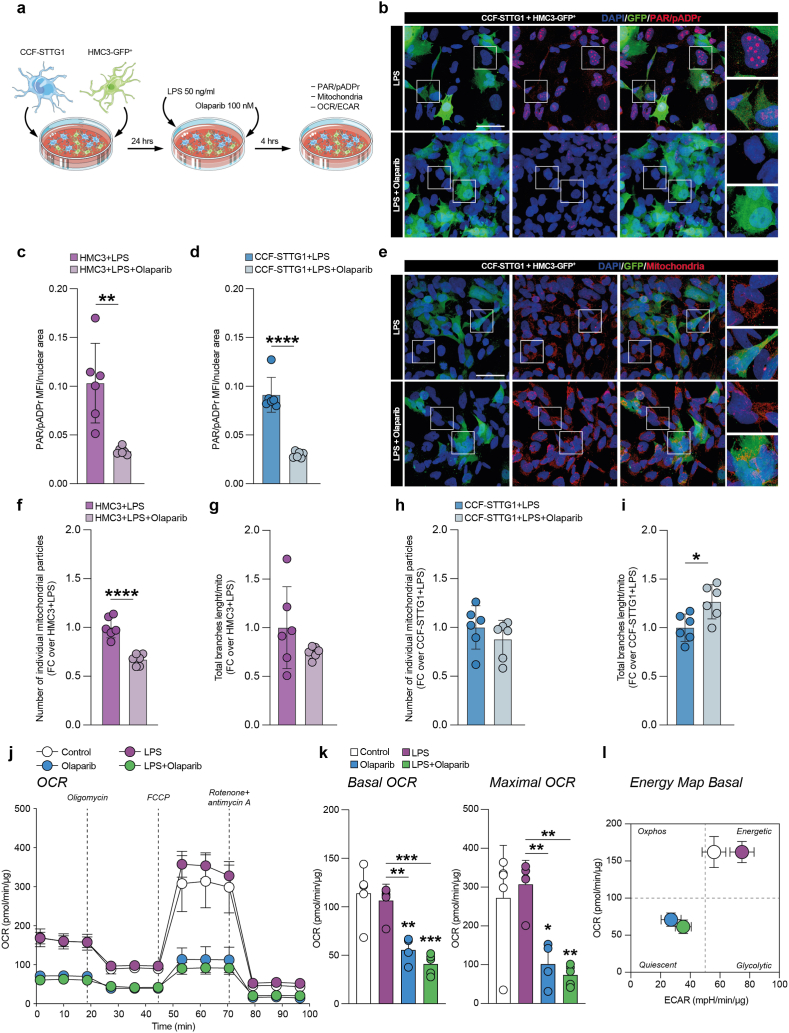
**Olaparib reduces PAR/pADPr accumulation and remodels mitochondrial morphology in an *in vitro* model of glial inflammation.** (a) Experimental design of the *in vitro* co-culture experiment. (b) Representative images of co-cultured HMC3 GFP^+^ (green) and CCF-STTG1 cells treated with LPS or LPS + Olaparib showing PAR/pADPr (red) staining. The white squares indicate regions at higher magnification shown on the right. Scale bar = 50 μm. (c) Quantification of the MFI of PAR/pADPr over the nuclear area in HMC3+LPS and HMC3+LPS + Olaparib cells. (d) Quantification of the MFI of PAR/pADPr in the nuclear area in the CCF-STTG1+LPS and CCF-STTG1+LPS + Olaparib groups. (e) Representative images of mitochondrial staining (red) in co-cultured HMC3 GFP^+^ (green) and CCF-STTG1 cells treated with LPS or LPS + Olaparib. The white squares indicate regions at higher magnification shown on the right. Scale bar = 50 μm. (f-g) Quantification of the number of individual mitochondrial particles and total branches length/mito in the HMC3+LPS and HMC3+LPS + Olaparib groups. (h-i) Quantification of the number of individual mitochondrial particles and total branches length/mito in the CCF-STTG1+LPS and CCF-STTG1+LPS + Olaparib groups. The data in (c-d and f-i) are shown as dot plot and means ± SDs of n = 6 biological replicates, and ∗p-value <0.05, ∗∗p-value <0.01, and ∗∗∗∗p-value <0.0001; two-tailed unpaired Student's *t*-test. (j) Normalized OCR of control, LPS, olaparib, and LPS + olaparib HMC3 and CCF-STTG1 co-cultures at 4 h, during sequential injections of 1.5 μM oligomycin, 1 μM FCCP, and 0.5 μM rotenone/antimycin A. The data in (j) are expressed as mean ± SEM of n ≥ 4 biological replicates. (k) Basal and maximal OCR of control, LPS, olaparib, and LPS + olaparib HMC3 and CCF-STTG1 co-cultures at 4 h. The data in (k) are expressed as dot plots and means ± SDs of n ≥ 4 biological replicates, and ∗p-value <0.05, ∗∗p-value <0.01, and ∗∗∗p-value <0.001 vs. control or between groups; one-way ANOVA followed by the Holm-Šídák multiple-comparison test. (l) Energy map biplot of basal OCR and ECAR for HMC3 and CCF-STTG1 co-cultures control, LPS, olaparib, and LPS + olaparib at 4 h. The data in (l) are expressed as mean ± SEM of n ≥ 4 biological replicates. ECAR: extracellular acidification rate, FC: fold change, LPS: lipopolysaccharide, MFI: mean fluorescence intensity, OCR: oxygen consumption rate.

Human microglial (i.e. HMC3-GFP^+^) and astrocyte-like (i.e. CCF-STTG1) cells were co-cultured and treated with olaparib following the experimental design shown in Fig. 1a. A final concentration of 50 ng/ml lipopolysaccharide (LPS) was chosen to selectively prime microglia, inducing a significant upregulation of the pro-inflammatory cytokine IL-1β (Fig. S1a). Notably, PARP1 inhibition with olaparib significantly reduced IL-1β expression without affecting basal levels in unstimulated cells, suggesting the inflammation-dependent activity of PARP1 (Fig. S1a). Immunofluorescence analyses revealed intense nuclear PAR/pADPr accumulation in LPS-stimulated HMC3-GFP^+^ cells, which was significantly attenuated by olaparib (Fig. 1b–c and Fig. S1b). Interestingly, co-cultured CCF-STTG1 cells presented a similar PAR/pADPr distribution pattern, suggesting a possible shift toward a reactive phenotype (Fig. 1b–d and Fig. S1c).

Given that PARP1 activation is linked to cellular metabolic stress, we next examined whether this response was associated with alterations in mitochondrial organization. Mitochondrial immunolabelling of co-cultured cells exposed to LPS (Fig. 1e) revealed that olaparib treatment significantly reduced the number of individual mitochondrial particles in HMC3-GFP^+^ cells without affecting mitochondrial branches length (Fig. 1f–g and Fig. S1d–e). In contrast, in co-cultured CCF-STTG1 cells, PARP1 inhibition did not affect the number of individual mitochondrial particles but significantly increased the average branches length (Fig. 1h–i and Fig. S1f–g).

To link the accumulation of PAR/pADPr and mitochondrial reshaping with functional metabolic shift induced by olaparib, we performed a XF assay of the OCR and ECAR during a mitochondrial stress test (Fig. 1j and Fig. S1h). We compared olaparib-treated and/or LPS stimulated co-cultures with untreated controls, finding that olaparib treatment significantly reduced basal and maximal OCR in HMC3 and CCF-STTG1 co-cultures at 4 h (Fig. 1k). Biplot analysis of OCR and ECAR showed that while LPS stimulation induced an early slight shift towards an energetic metabolic profile in LPS-stimulated co-cultures, olaparib invariably led to a quiescent phenotype in control and LPS-stimulated HMC3 and CCF-STTG1 co-cultures (Fig. 1l). These data indicate that PARP1 inhibition affects inflammatory signalling via mitochondrial network and cell metabolism reshaping in glial cells.

**Fig. 2:**
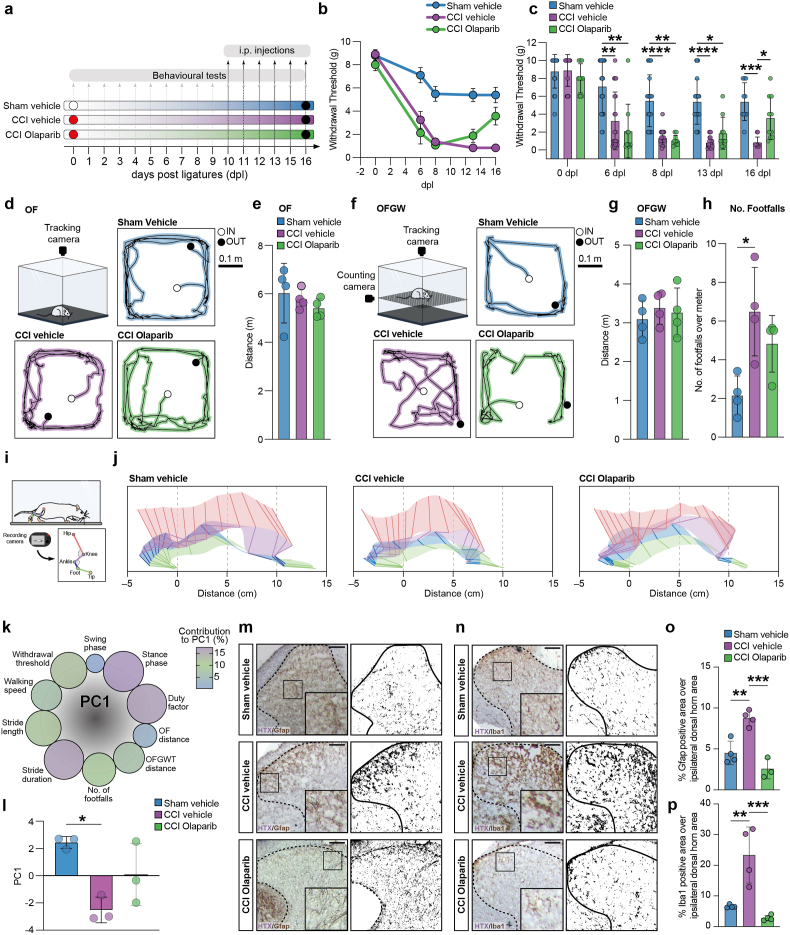
**Inhibition of PARP1 activity attenuates neuropathic pain and spinal gliosis in CCI rats.** (a) Schematic representation of the experimental timeline for surgeries, behavioural tests and treatments. The red dots indicate CCI surgery, and the black dots indicate the final time-points for neuropathological analyses. (b) Time-course of paw withdrawal thresholds measured by von Frey filaments in sham vehicle-, CCI vehicle- and CCI olaparib-treated rats. (c) Repeated measures of withdrawal thresholds at 0, 6, 8, 13 and 16 dpl in the sham vehicle, CCI vehicle and CCI olaparib groups. The data in (b-c) are shown as dot/dot plots and means ± SDs of n ≥ 10 rats per group, and ∗p-value <0.05, ∗∗p-value <0.01, ∗∗∗p-value <0.001, and ∗∗∗∗p-value <0.0001; mixed-effects two-way ANOVA followed by the Holm-Šídák multiple-comparison test. (d) Experimental setup for the OF test and representative tracks of the sham vehicle, CCI vehicle and CCI olaparib groups at 16 dpl. (e) Quantification of the total distance travelled during OF in the sham vehicle, CCI vehicle and CCI olaparib groups. (f) Experimental setup for the OFGW test and representative tracks of the sham vehicle, CCI vehicle and CCI olaparib groups. (g) Quantification of the total distance travelled during OFGW in the sham vehicle, CCI vehicle and CCI olaparib groups at 16 dpl. (h) Quantification of the number of footfall errors in the OFGW test. The data in (e, g-h) are shown as dot plots and means ± SDs of n = 4 animals per group, and ∗p-value <0.05; one-way ANOVA followed by the Holm-Šídák multiple-comparison test. (i) Experimental setup for the kinematic assessment of the sham vehicle, CCI vehicle and CCI olaparib groups. (j) Representative color-coded stick view decomposition of the rats left hindlimb movement during stepping of sham vehicle, CCI vehicle, and CCI olaparib rats. (k) Random circular bubble plot of the relative contribution of measured variables to PC1 derived from principal component analysis (PCA). Circle size is proportional to the contribution of the variable to PC1, and colour intensity indicates the contribution as percentage. (l) Composite score expressed as PC1 value of the sham vehicle, CCI vehicle and CCI olaparib groups. The data in (l) are shown as dot plots and means ± SDs of n = 3 animals per group, and ∗p-value <0.05; one-way ANOVA followed by the Holm-Šídák multiple-comparison test. (m-p) Representative images of Gfap (m) and Iba1 (n) immunostaining, and quantification of the Gfap-positive (o) and Iba1-positive (p) immunoreactive area expressed as a percentage of the ipsilateral dorsal horn area in the sham vehicle, CCI vehicle and CCI olaparib groups. Insets highlight high-magnification areas of boxed regions. Scale bars = 100 μm. The data in (o-p) are shown as dot plots and means ± SDs of n ≥ 3 rats per group, and ∗∗p-value <0.01, ∗∗∗p-value <0.001; one-way ANOVA followed by the Holm-Šídák multiple-comparison test. Dpl: days post-ligatures, i.p.: intraperitoneal, PC1: principal component 1; OF: open field, OFGW: open field grid walk. HTX: hematoxylin.

**Olaparib attenuates mechanical allodynia, suppresses spinal gliosis, and redirects cell death pathways in CCI rats.** Given the extensive reactive gliosis observed in chronic neuropathic pain [[23], [24], [25]], and building on the *in vitro* evidence of PARP1-dependent inflammatory dysregulation, we next assessed whether PARP1 inhibition mediated by olaparib could counteract mechanical allodynia *in vivo*. To this end, we employed the CCI model, which via four ligatures tied around the sciatic nerve reliably induces mechanical allodynia, maladaptive plasticity in the CNS and persistent gliosis in the dorsal horn of the spinal cord [[26], [27], [28]]. Mechanical hypersensitivity was assessed over a 16-day window following sham and CCI surgeries (Fig. 2a). CCI induced a progressive and persistent reduction in paw withdrawal thresholds, which reached a final plateau between 8 and 13 days post-ligatures (dpl) (Fig. 2b and c). At this stage, when the neuropathy was fully developed (i.e., 10 dpl), a daily intraperitoneal (i.p.) injection of vehicle or olaparib was started (Fig. 2a). Compared with CCI vehicle rats, CCI rats treated with olaparib exhibited significant attenuation of mechanical allodynia, as evidenced by improved thresholds at 16 dpl (Fig. 2b and c). To determine whether this improvement was accompanied by an amelioration of locomotor behaviour and motor coordination, the rats were tested in the open field (OF) and open field grid walking (OFGW) settings (Fig. 2d–h). Locomotor exploration, quantified as the total distance travelled, was comparable across all groups, indicating that olaparib did not exert any sedative or motor-suppressive effects (Fig. 2e and g). However, OFGW analyses revealed a significant increase in the total number of footfalls in CCI rats compared with sham controls. Notably, olaparib-treated animals presented a reduction in footfall errors, suggesting a partial rescue of motor coordination (Fig. 2h). Analysis of Nissl-stained spinal cord samples showed no evident signs of motoneuronal suffering or depletion, nor morphological changes associated with chromatolytic appearance, typically associated with reduction of Nissl bodies staining and peripherally located nuclei (Fig. S2a). To deeply assess motor performance in CCI vehicle rats and CCI rats treated with olaparib compared with sham controls, we also performed a comprehensive kinematic analysis (Fig. 2i–j and Fig. S2b–i). We observed marked changes in knee and ankle angle during stepping in CCI vehicle rats compared to sham controls, coupled with slight alterations in the overall kinematic and gait parameters (Fig. S2b–i). Our data also showed a significant reduction of the stride length in CCI vehicle rats, coherent with allodynia and increased pain perception, partially reverted by olaparib administration (Fig. S2e). We finally generated a composite score using a principal component analysis (PCA) on all tested behavioural and functional outcomes (Fig. 2k). PC1 as a composite score, explaining 54.9% of the variances (Fig. S2j), was significantly reduced in CCI vehicle rats as compared to sham vehicle and this phenomenon was reverted by CCI olaparib treatment (Fig. 2l). These results indicate that PARP1 inhibition can reverse established CCI-associated behaviour *in vivo*, prompting further investigation into the involved cellular and molecular mechanisms.

Neuroinflammation in neuropathic pain is sustained by glial reactivity within the spinal cord dorsal horn, where both astrocytes and microglia contribute to the amplification of nociceptive signalling [23]. To assess whether the activity of PARP1 inhibition *in vivo* mirrors the glial responses observed *in vitro*, we quantified the proportions of Iba1-and Gfap-positive cells in the dorsal horns of the spinal cord (Fig. 2m and n). By analysing the percentage of Gfap-positive area, we found a significant increase following CCI, which was markedly reduced by olaparib treatment (Fig. 2o). Similarly, the Iba1-positive signal was strongly elevated in vehicle-treated CCI rats, whereas PARP1 inhibition significantly reversed this upregulation (Fig. 2p).

The expression of connexin 43 (Cx43), whose upregulation has been consistently associated with maladaptive glial communication in the context of neuropathic pain [27], was also evaluated (Fig. S3a). Notably, confocal imaging revealed a pronounced glial activation profile in CCI animals, characterized by dense and overlapping Gfap/Iba1-positive signals with increased Cx43-associated peaks in both astrocytic and microglial compartments, features that were markedly attenuated by olaparib (Fig. S3b). Moreover, morphometric analyses of microglia revealed that although no significant differences in the area or perimeter parameters of Iba1-positive cells were detected, olaparib induced a significant increase in microglial circularity (Fig. S3c). To better understand the microglial profile upon nerve injury and PARP1 inhibition, we next assessed the expression of arginase-1 (Arg-1), a metabolic enzyme associated with inflammation-resolving programs in myeloid cells [29]. Our data revealed a marked increase in Arg1 expression in the spinal cord of the CCI olaparib-treated animals compared with the CCI vehicle-treated and sham vehicle-treated animals (Fig. S3d). We also tested Chi3L1 expression, as a marker of maladaptive astrogliosis, in the sham vehicle, CCI vehicle and CCI olaparib spinal cord dorsal laminae, finding a significant increase in Chi3L1 MFI in CCI rats, fully reverted by olaparib treatment (Fig. S3e–f). This evidence was coupled with a significant colocalization coefficient with Gfap, thus indicating an olaparib-induced reshaping in astrocyte phenotype (Fig. S3g). Together with the previous findings, these observations suggest that PARP1 inhibition not only dampens reactive microglia but also may reduce a shift toward a maladaptive phenotype.

**Fig. 3:**
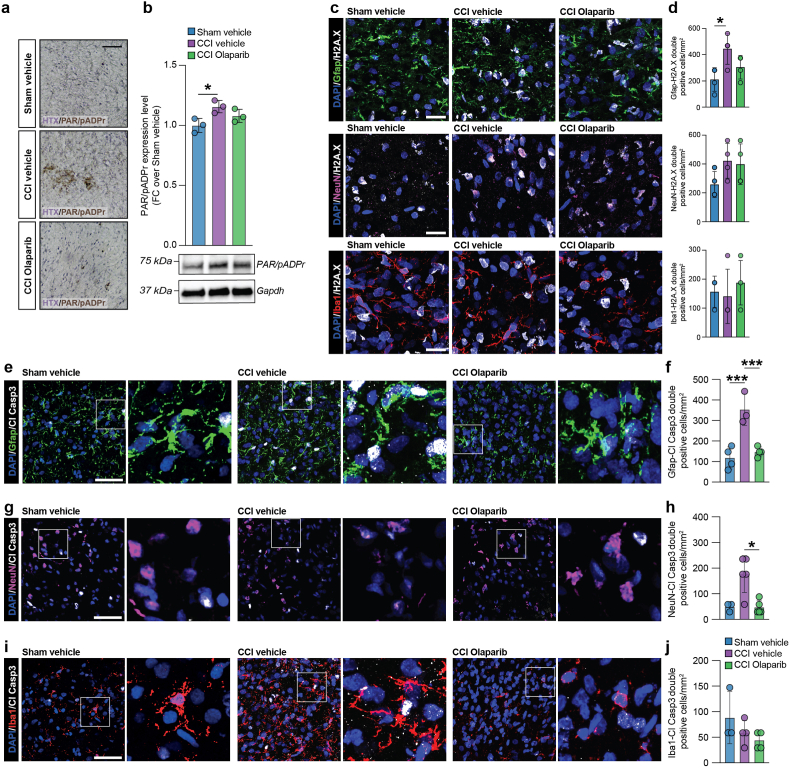
**Olaparib-mediated Parp1 inhibition reduces PAR polymers accumulation and cell death in the spinal cord.** (a) Representative images showing PAR/pADPr signals in spinal cord sections from the sham vehicle, CCI vehicle and CCI olaparib groups. Scale bar = 50 μm. (b) Quantification and representative blot of PAR/pADPr expression levels in spinal cord lysates from the sham vehicle, CCI vehicle and CCI olaparib groups. (c) Representative images of H2A.X (white)- and Gfap (green)-, NeuN (purple)-, or Iba1 (red)-positive cells in the spinal cord dorsal horn sections. (d) Quantification of Gfap-H2A.X, NeuN-H2A.X, and Iba1-H2A.X double positive cells in sham vehicle, CCI vehicle and CCI olaparib rats. (e) Representative images of Cl Casp3 (white)- and Gfap (green)-positive cells in the spinal dorsal horn sections. (f) Quantification of Cl Casp3-Gfap double-positive cells in sham vehicle, CCI vehicle and CCI olaparib rats. (g) Representative images of Cl Casp3 (white)- and NeuN (purple)-positive cells in the spinal dorsal horn sections. (h) Quantification of Cl Casp3-NeuN double-positive cells in sham vehicle, CCI vehicle and CCI olaparib rats. (i) Confocal-assisted representative images of Cl Casp3 (white)- and Iba1 (red)-positive cells in the spinal dorsal horn sections. (j) Quantification of Cl Casp3-Iba1 double-positive cells in sham vehicle, CCI vehicle and CCI olaparib rats. The white boxes indicate the magnified areas shown on the right. Scale bars = 50 μm. The data are shown as dot plots and means ± SDs of n ≥ 3 animals per group, and ∗p-value <0.05, ∗∗∗p-value <0.001; one-way ANOVA followed by the Holm-Šídák multiple-comparison test (b, d and f) and Kruskal-Wallis followed by the Dunn's test for multiple-comparisons (h and j).

To test the hypothesis that PARP1 hyperactivation underlies the observed neuroinflammatory changes, we examined the accumulation of PAR polymers at the spinal level as a direct indicator of PARP1 activity. As shown by immunostaining and confirmed via western blotting, elevated PAR/pADPr levels were detected in the spinal cords of CCI rats, and were partially reduced following PARP1 inhibition (Fig. 3a and b).

To explore whether this PARP1 hyperactivity was correlated with an early event in parthanatos, we assessed DNA damage via H2A.X immunostaining, a marker of double-strand DNA breaks, in neurons, astrocytes, and microglia (Fig. 3c and d). CCI vehicle spinal cord sections presented a marked increase in H2A.X-Gfap double-positive cells within the dorsal horn, indicating the presence of unresolved genotoxic stress consistent with sustained PARP1 activity (Fig. 3c and d). This effect was abolished in the animals treated with olaparib (Fig. 3c and d). Interestingly, while the proportion of H2A.X-NeuN double-positive cells was slightly, although not significantly, increased in CCI rats, H2A.X-Iba1 double-positive cells remained unchanged across conditions (Fig. 3c and d).

Given that parthanatos is a caspase-independent cell death pathway, we also examined cleaved caspase 3 (Cl Casp3) expression in neurons, astrocytes, and microglia to better characterize the affected cell population and to delineate the involvement of parallel apoptotic mechanisms. Co-immunostaining of Cl Casp3 with Gfap revealed a robust increase in Cl Casp3-positive astrocytes in the spinal dorsal horn of CCI rats, which was significantly reduced by olaparib treatment (Fig. 3e and f). Notably, the proportion of NeuN-positive cells displayed a similar trend, with the number of NeuN-Cl Casp3 double positive cells significantly reduced by olaparib (Fig. 3g and h). In contrast, the proportion of Cl Casp3-Iba1 double-positive cells remained unchanged across conditions (Fig. 3i and j). These different patterns suggest that microglia may represent a target cell population involved in comprehensive reshaping of spinal cord microenvironment in neuropathic pain.

**Fig. 4:**
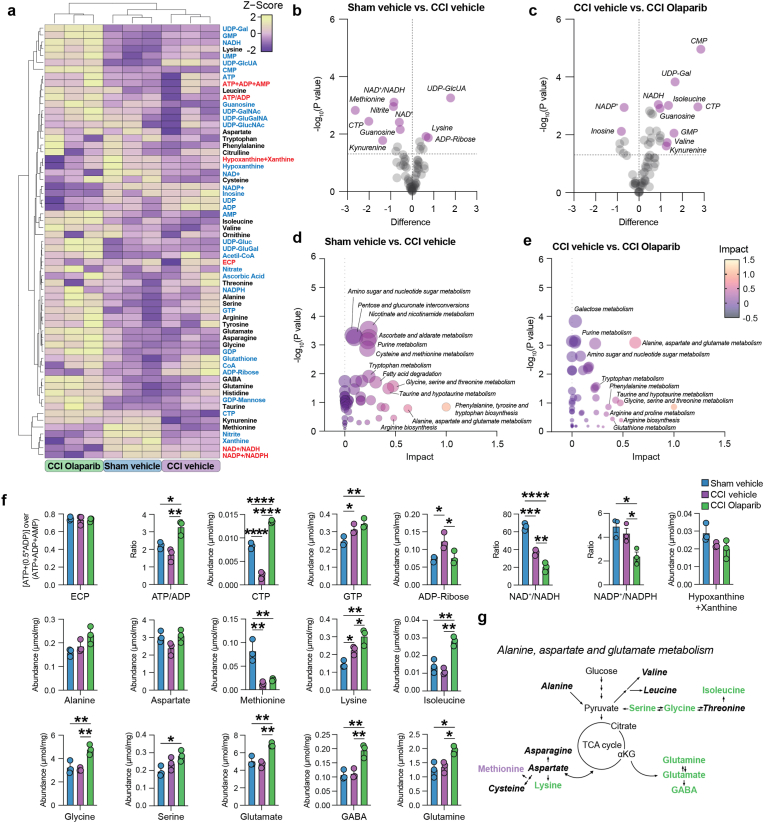
**Olaparib reshapes the spinal cord metabolic signature of CCI.** (a) Heatmap and cluster analyses of 57 metabolites and 6 derived values (sums, ratios, and products) across sham vehicle, CCI vehicle and CCI olaparib spinal cord samples. The data are shown as z-score normalized values of absolute metabolite abundance. Metabolites are color labelled according to the functional class: energetic metabolites (blue), amino acids (black) and metabolite ratios (red). (b-c) Volcano plot analysis of spinal cord metabolites expressed as – log_10_ of p-value comparing the sham vs. CCI vehicle (b) and CCI vehicle vs. CCI olaparib (c) groups. The purple dots indicate significantly modulated metabolites. (d-e) Data enrichment analyses with the most statistically and biologically relevant pathways in the sham vehicle vs. CCI vehicle (d) and CCI vehicle vs. CCI olaparib (e) groups. Dots are key-colored according to their impact. (f) Abundance (μmol/mg) of selected metabolites. The data are shown as dot plots and means ± SDs of n = 3 rats per group, and ∗p-value <0.05, ∗∗p-value <0.01, ∗∗∗p-value <0.001, ∗∗∗∗p-value <0.0001; one-way ANOVA followed by the Holm-Šídák multiple-comparison test. (g) Schematic representation of the alanine, aspartate and glutamate metabolism pathway, with reduced amino acids in CCI olaparib in purple and increased amino acids in CCI olaparib in green.

**PARP1 inhibition modulates metabolic pathways associated with energy balance**. To investigate whether these changes extended to core metabolic circuits, we next conducted a targeted metabolic analysis on spinal cord tissues, focusing on phosphate nucleotides, nicotinic coenzymes and amino acids (Fig. 4a and Fig. S4a–d). Volcano plot analysis showed robust metabolic reprogramming induced by nerve injury and by olaparib treatment (Fig. 4b and c). Pathway enrichment analysis revealed that glutamate, alanine-aspartate and purine metabolism were the most dysregulated networks driven by CCI (Fig. 4d–e and Fig. S4e). Compared with CCI olaparib, CCI vehicle resulted in reduced ATP/ADP ratio and accumulation of ADP-ribose, which is consistent with the metabolic exhaustion driven by PARP1 hyperactivation (Fig. 4f and Fig. S5). In parallel, the modulation of metabolites such as lysine, isoleucine, glycine, serine, glutamate, gamma-aminobutyric acid (GABA), and glutamine suggests a broader reorganization of intermediary amino acid metabolic nodes, reflecting a strong effect on mitochondrial function and energy balance (Fig. 4f and g).

**Fig. 5:**
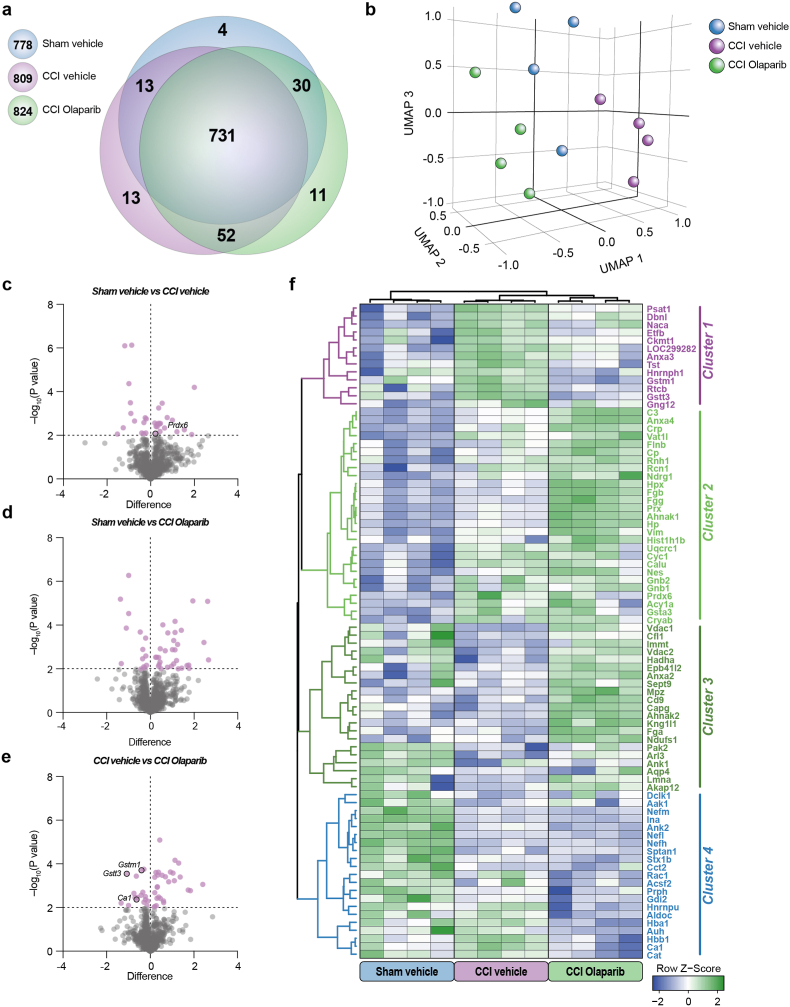
**Olaparib modulates proteomic profile in the spinal cord of CCI rats.** (a) Venn diagram representing the total number of proteins identified across all experimental conditions, highlighting the shared and condition-specific proteins. (b) 3D UMAP of the total proteome. Each point represents a biological replicate. (c-e) Volcano plots of total analysed proteins in the indicated pairwise comparisons, expressed as the –log_10_ of p-value. The purple dots indicate significantly modulated proteins under each condition. (f) Hierarchical clustering of differentially expressed proteins and heatmap of z-score values based on the abundance of 82 proteins significantly modulated. The data include n = 4 biological replicates per group.

**Spinal proteomic profile reveals redox-related proteins modulation as downstream effect of PARP1 inhibition**. To further characterize the molecular mechanisms underlying the efficacy of PARP1 inhibition and neuroprotective effects following nerve injury, we performed an untargeted quantitative proteomic analysis of lumbar spinal cord tissues. Unbiased clustering revealed a highly conserved core of 731 commonly expressed proteins across all conditions, alongside specific subsets altered by injury or by olaparib treatment (Fig. 5a). 3D UMAP clustering showed a clear shift induced by CCI that was partially reversed following olaparib treatment, resulting in a closer cluster toward the sham profile (Fig. 5b). Differential expression analyses identified a set of proteins significantly altered by CCI, many of which were modulated by PARP1 inhibition (Fig. 5c–e).

**Fig. 6:**
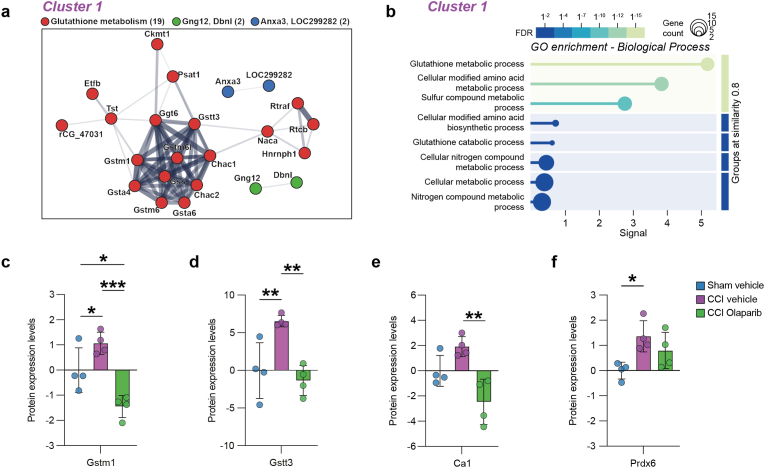
**Olaparib modulates redox and cellular stress adaptation in the spinal cord of CCI rats.** (a) Interaction network of significantly modulated proteins in cluster 1 and the top interacting genes; interaction thickness indicates the strength of data support. (b) Functional enrichment gene ontology (GO) analysis of biological processes associated with proteins in cluster 1. (c-f) Relative abundance of Gstm1 (c), Gstt3 (d), Ca1 (e), and Prdx6 (f) protein levels showing injury-induced changes and olaparib effects. The data are shown as dot plots and means ± SDs of n = 4 animals per group. One-way ANOVA followed by the Holm-Šídák multiple-comparison test, and ∗p-value <0.05, ∗∗p-value <0.01, ∗∗∗p-value <0.001; one-way ANOVA followed by the Holm-Šídák multiple-comparison test. FDR: false discovery rate.

To better visualize the distribution of differentially expressed proteins, we generated an unsupervised hierarchical heatmap based exclusively on significant hits from the pairwise comparisons (Fig. 5f). Our analysis revealed a distinct grouping of samples and highlighted 4 clusters of proteins whose expression was increased by CCI and reversed by PARP1 inhibition (cluster 1), modulated by olaparib (clusters 2 and 3) or reduced in both the CCI vehicle and CCI olaparib groups (cluster 4). The interaction network of significantly modulated proteins associated with cluster 1 revealed a robust network of interactions among proteins involved in glutathione metabolism (Fig. 6a). The functional enrichment analysis of the biological processes associated with cluster 1 consistently showed that the glutathione metabolic process was the most significant one, together with the cellular modified amino acid metabolic process (Fig. 6b). The significantly modulated proteins of clusters 2-4 were involved mainly in mixed biological processes, such as acute-phase response (i.e. cluster 2), mitochondrial membrane organization and mitochondrion organization (i.e. cluster 3), and hydrogen peroxide catabolic processes (i.e. cluster 4; Fig. S6). Given the involvement of key regulators of oxidative stress and detoxification, among the CCI-induced upregulated proteins, glutathione S-transferase Mu 1 (Gstm1), glutathione S-transferase theta 3 (Gstt3), and carbonic anhydrase 1 (Ca1) were significantly reverted by olaparib (Fig. 6c–e). We also found that the expression of peroxiredoxin 6 (Prdx6), which was increased in CCI rats, was restored by PARP1 inhibition (Fig. 6f). Collectively, these findings point to a widespread remodelling of the injury-induced molecular programs, particularly to those linked to redox control and cellular stress adaptation.

**Fig. 7:**
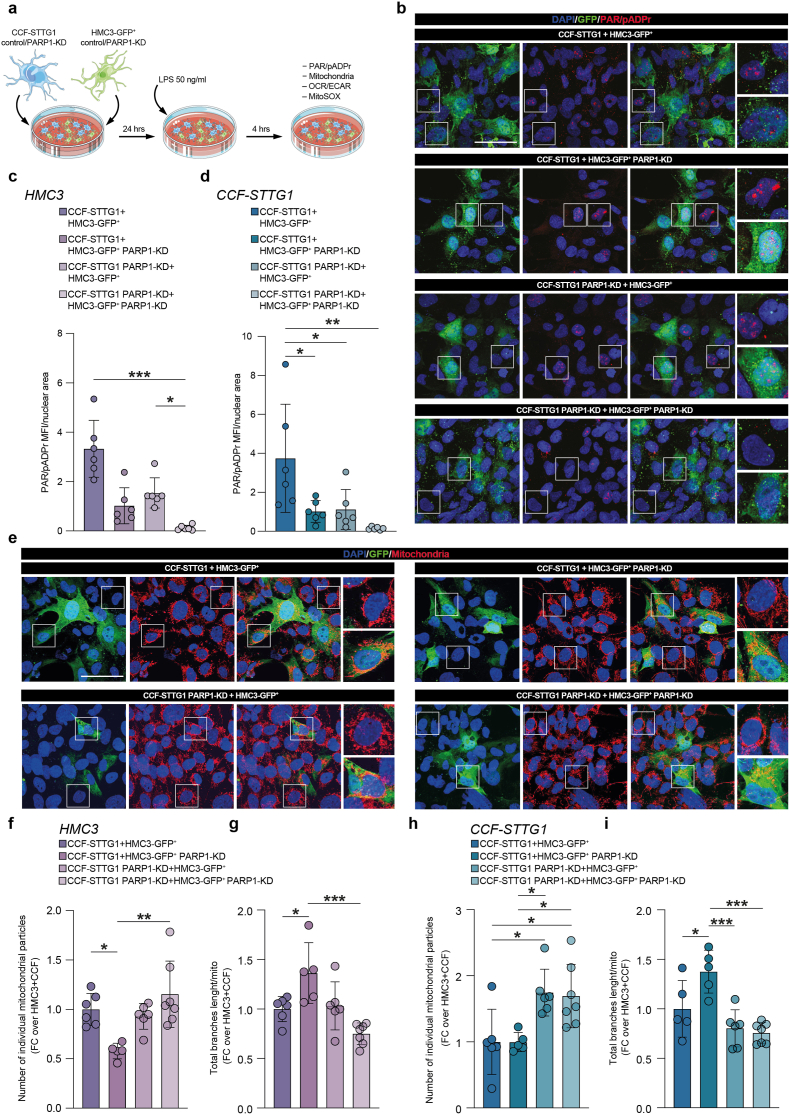
**Cell-type specific PARP1 knockdown reduces PAR/pADPr accumulation and remodels mitochondrial morphology in neuroglia co-cultures.** (a) Experimental design of the *in vitro* control/PARP1-knockdown (KD) co-culture experiment. (b) Representative images of LPS treated co-cultures of HMC3-GFP^+^/HMC3-GFP^+^ PARP1-KD (green) and/or CCF-STTG1/CCF-STTG1 PARP1-KD cells showing PAR/pADPr (red) staining. The white squares indicate regions at higher magnification shown on the right. Scale bar = 50 μm. (c-d) Quantification of PAR/pADPr MFI over the nuclear area in HMC3-GFP^+^/HMC3-GFP^+^ PARP1-KD cells (c) or in CCF-STTG1/CCF-STTG1 PARP1-KD cells (d) in co-cultures. (e) Representative images of mitochondrial staining (red) in LPS treated co-cultures of HMC3-GFP^+^/HMC3-GFP^+^ PARP1-KD and/or CCF-STTG1/CCF-STTG1 PARP1-KD cells. The white squares indicate regions at higher magnification shown on the right. Scale bar = 50 μm. (f-g) Quantification of number of individual mitochondrial particles (f) and total branches length/mito (g) in HMC3-GFP^+^/HMC3-GFP^+^ PARP1-KD from LPS treated co-cultures of HMC3-GFP^+^/HMC3-GFP^+^ PARP1-KD and/or CCF-STTG1/CCF-STTG1 PARP1-KD cells. (h-i) Quantification of number of individual mitochondrial particles (h) and total branches length/mito (i) in CCF-STTG1/CCF-STTG1 PARP1-KD from LPS treated co-cultures of HMC3-GFP^+^/HMC3-GFP^+^ PARP1-KD and/or CCF-STTG1/CCF-STTG1 PARP1-KD cells. The data are shown as dot plots and means ± SDs of n ≥ 5 biological replicates, and ∗p-value <0.05, ∗∗p-value <0.01, ∗∗∗p-value <0.001; one-way ANOVA followed by the Holm-Šídák multiple-comparison test. FC: fold change, MFI: mean fluorescence intensity.

**Cell-type specific PARP1 knockdown reshapes mitochondrial metabolism and redox balance in neuroglia co-cultures.** To validate the causal link between PARP1 inhibition and cell-type specific effects, we went back to the heterocellular co-culture system of HMC3-GFP^+^ and CCF-STTG1 using either microglia and/or astrocyte *PARP1*-knockdown (KD, Fig. 7a and Fig. S7a–b). We primed HMC3-GFP^+^ or HMC3-GFP^+^ PARP1-KD cells using LPS and performed a quantification of PAR/pADPr nuclear accumulation (Fig. 7b–c and Fig. S7c–d). We observed that all PARP1-KD co-cultures showed a reduction of nuclear PAR/pADPr in microglia- and astrocyte-like cells, with a substantial reduction in HMC3-GFP^+^ PARP1-KD and CCF-STTG1 PARP1-KD co-cultures (Fig. 7b–d and Fig. S7c–d). We also examined whether mitochondrial parameters were similarly affected by PARP-KD (Fig. 7e and Fig. S7e–f). Our data confirmed that PARP1-KD in microglia reduced the number of individual mitochondrial particles (Fig. 7f and Fig. S7e) with a slight increase in the total branches length per mito (Fig. 7g and Fig. S7e). Interestingly, we observed that co-cultures between HMC3-GFP^+^ PARP1-KD and CCF-STTG1 showed unchanged number of individual mitochondrial particles in CCF-STTG1 (Fig. 7h and Fig. S7f), with a significant increase in the total branches length per mito (Fig. 7i and Fig. S7f), similarly to olaparib-induced effects, thus indicating that microglia PARP1 activity represents a key driver of changes associated with LPS priming.

Whether CCF-STTG1-restricted PARP1-KD showed no significant effects on HMC3-GFP^+^ (Fig. 7f and g), we found that the total number of individual mitochondrial particles was increased in CCF-STTG1 (Fig. 7h), and full PARP1-KD co-cultures showed reduced branches length per mito in HMC3-GFP^+^ and increased number of individual particles in CCF-STTG1 (Fig. 7f–i and Fig. S7e-f).

**Fig. 8:**
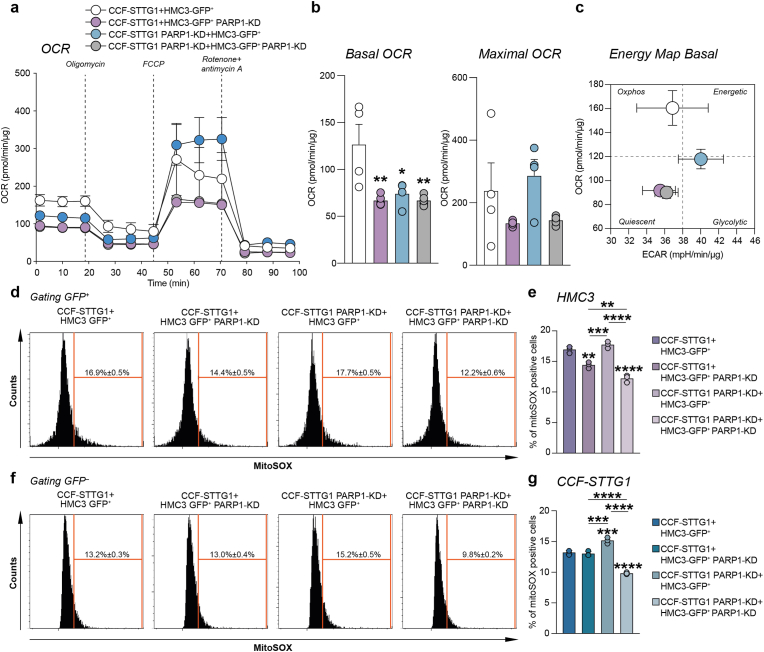
**Cell-type specific PARP1 knockdown induces quiescent phenotype and reduces mitochondrial ROS in neuroglia co-cultures.** (a) Normalized OCR of LPS treated co-cultures of HMC3-GFP^+^/HMC3-GFP^+^ PARP1-KD and/or CCF-STTG1/CCF-STTG1 PARP1-KD cells at 4 h, during sequential injections of 1.5 μM oligomycin, 1 μM FCCP, and 0.5 μM rotenone/antimycin A. The data are expressed as mean ± SEM of n ≥ 4 biological replicates. (b) Basal and maximal OCR of LPS treated co-cultures of HMC3-GFP^+^/HMC3-GFP^+^ PARP1-KD and/or CCF-STTG1/CCF-STTG1 PARP1-KD cells at 4 h. The data are expressed as means ± SDs of n ≥ 4 biological replicates, and ∗p-value <0.05 and ∗∗p-value <0.01 vs. CCF-STTG1+HMC3-GFP^+^; one-way ANOVA followed by the Holm-Šídák multiple-comparison test. (c) Energy map biplot of basal OCR and ECAR for LPS treated co-cultures of HMC3-GFP^+^/HMC3-GFP^+^ PARP1-KD and/or CCF-STTG1/CCF-STTG1 PARP1-KD cells at 4 h. Data are expressed as mean ± SEM of n ≥ 4 biological replicates. (d-e) Representative mitoSOX histograms (d) and quantification (e) of cytofluorimetric analysis gating GFP^+^ HMC3-GFP^+^/HMC3-GFP^+^ PARP1-KD cells from LPS treated co-cultures of HMC3-GFP^+^/HMC3-GFP^+^ PARP1-KD and/or CCF-STTG1/CCF-STTG1 PARP1-KD cells. (f-g) Representative mitoSOX histograms (f) and quantification (g) of cytofluorimetric analysis gating GFP^–^ CCF-STTG1/CCF-STTG1 PARP1-KD from LPS treated co-cultures of HMC3-GFP^+^/HMC3-GFP^+^ PARP1-KD and/or CCF-STTG1/CCF-STTG1 PARP1-KD cells. Data in e and g are shown as a % gated cells and shown as dot plots and means ± SDs of n = 3 independent replicates. ∗p-value <0.05, ∗∗p-value <0.01, ∗∗∗p-value <0.001, and ∗∗∗∗p-value <0.0001 vs CCF-STTG1+HMC3-GFP^+^ or between groups; one-way ANOVA followed by the Holm-Šídák multiple-comparison test. OCR: oxygen consumption rate, ECAR: extracellular acidification rate.

To functionally assess the effects of cell-type specific PARP1-KD, we performed a mitochondrial stress test analysing the OCR and ECAR in HMC3-GFP^+^ PARP1-KD and CCF-STTG1 PARP1-KD co-cultures (Fig. 8a and Fig. S8a). Our data showed a significant reduction of the basal OCR in all tested PARP1-KD co-cultures, coupled with a slight, although not significant, reduction of maximal OCR in co-cultures with HMC3 PARP1-KD (Fig. 8b). Biplot analysis of OCR and ECAR showed that PARP1-KD on microglia induces an early shift towards a quiescent metabolism in microglia-astrocytes co-cultures (Fig. 8c), coherent with olaparib-induced effects.

In an effort to link this evidence with redox balance observed in the *in vivo* experiments, we performed a cytofluorimetric-assisted quantification of mitochondrial ROS using mitoSOX staining in CCF-STTG1 and HMC3-GFP^+^ co-cultures with cell-type specific PARP1-KD (Fig. 8d–g and Fig. S8b–d). Our data showed that HMC3-GFP^+^ PARP1-KD induced a significant reduction of mitoSOX in HMC3-GFP^+^ (Fig. 8f and g), while co-cultures with CCF-STTG1-restricted PARP1-KD showed a slight increase in mitoSOX (Fig. 8f and g), compatible with ROS ability to act as signalling molecules. Interestingly, no significant effects were observed in HMC3-GFP^+^ cells co-cultured with CCF-STTG1 PARP1-KD cells (Fig. 8f and g). Importantly, co-cultures with full PARP1-KD showed a significant reduction of mitoSOX in both astrocyte- and microglia-like cells (Fig. 8d–g). These findings support an extensive remodelling induced by PARP1-KD, associated with mitochondrial metabolism and redox balance, pointing towards a microglia-associated cellular stress adaptation influencing astrocyte homeostasis and signalling.

## 3 Discussion

Neuropathic pain remains one of the most therapeutically intractable forms of chronic pain, mainly due to its multifactorial origin and the maladaptive reprogramming of neuronal and glial networks [30]. Current therapies, which are largely symptomatic, fail to address the molecular drivers of pain persistence. Our findings position PARP1 as a key factor in inflammatory and metabolic dysfunction in the spinal cord, expanding beyond its role in DNA repair and cell death, toward the active shaping of the neuropathic microenvironment [31]. Although PARP1 activity has been extensively studied in oncology, its contribution to neuroimmune signalling in chronic pain has remained largely unexplored. The capacity of glial cells to integrate immune signals and remodel the local environment in response to nerve injury is central to the pathogenesis of neuropathic pain.

To explore the role of PARP1 in modulating the glial response to inflammatory stimuli, we used a reductionistic *in vitro* co-culture model of microglia and astrocytes. The selective priming of HMC3 cells with LPS and olaparib in combination with CCF-STTG1 cells significantly reduced PAR polymers accumulation in both HMC3 and CCF-STTG1 cell types. We further examined whether PARP1 hyperactivation is linked to mitochondrial stress, a key feature of neuroinflammation and glial dysfunction. In LPS-stimulated HMC3 cells, we observed a reduction in the number of individual mitochondrial particles following olaparib treatment. This effect was accompanied by changes in CCF-STTG1 cells, which presented increased mitochondrial branches length, suggesting that the network remodelled toward a more interconnected and potentially resilient state [22]. These findings align with previous evidence linking PARP1 hyperactivation to impaired mitochondrial bioenergetics, at least partially through disruption of mitochondrial complex I function. In contrast, pharmacological inhibition or genetic deletion of PARP1 restored NAD^+^ levels, ATP production and overall mitochondrial stabilization [[32], [33], [34]]. Previous reports have indicated that treatment with PJ34, a PARP1 inhibitor, increases the expression of genes involved in both mitochondrial fusion and fission in PC12 cells, suggesting that PARP1 inhibition promotes broad remodelling of mitochondrial dynamics [35].

To determine whether modulating glial inflammatory tone and mitochondrial dysfunction can ameliorate chronic pain *in vivo*, we evaluated the role of PARP1 in a model of neuropathic pain. Olaparib significantly alleviated mechanical allodynia and improved motor coordination without inducing any visible reduction in the distance covered during the OF and OFGW tests. Moreover, our behavioural analysis points towards an improved motor performance associated with reduced pain-related motor interference, with no evident sign of motoneuron degeneration and/or compensatory processes. These data are coherent with the involvement of broad spinal cord-resident cell populations rather than a cell-autonomous motoneuronal effect. Indeed, it appears to emerge from the restoration of the spinal cord microenvironment, where neuroinflammation, redox imbalance and metabolic collapse converge to maintain central sensitization [36,37].

Reactive gliosis and increased intercellular communication have been associated with the chronic phase of neuropathic pain [23,27,28]. Our data support an increase in pro-apoptotic signalling in the sensory laminae of the spinal cord in CCI rats, which is consistent with a loss of neuroglia homeostatic functions, the onset of stressful stimuli and progressive neuronal loss. Previous findings have shown that sustained pro-apoptotic signalling coexists with ROS accumulation [38,39], and this phenomenon is coherent with the increased PAR polymers observed in CCI rats and the increased proportion of H2A.X positive nuclei, particularly on astrocytes. This evidence is in accordance with a sustained maladaptive gliosis in CCI and a beneficial reshaping of astroglial phenotype mediated by PARP1 inhibition. Interestingly, we did not observe evident pro-apoptotic signalling and H2A.X nuclear accumulation in microglia mediated by chronic neuropathic pain. However, morphological remodelling and a shift toward a neuroprotective and phagocytic phenotype were observed upon PARP1 inhibition, indicating functional reprogramming rather than cytotoxicity. This effect on microglia cells is supported by previous studies showing that PARP1 inhibition reduces pro-inflammatory genes expression in reactive microglia without impairing their viability [40,41]. Moreover, PARP1 genetic deletion was shown to prevent the release of neurotoxic mediators by reactive microglia, thereby protecting neurons from secondary injury [42]. These findings support the idea that PARP1 inhibition promotes the functional reprogramming of microglia toward a neuroprotective and reparative state.

To further investigate the downstream effects of PARP1 inhibition underlying behavioural amelioration and neuroglial modulation, we performed targeted metabolic profiling and unbiased proteomic analysis of spinal cord tissues from sham and CCI rats. The accumulation of PAR polymers in the CCI spinal cord was consistent with increased PARP1 enzymatic activity and was reduced following olaparib treatment. Here, we also show, for the first time, evidence of the accumulation of monomeric ADP-ribose in chronic pain, which reflects both PAR catabolism and direct NAD^+^ hydrolysis [43]. We observed that PARP1 inhibition restored ADP-ribose to physiological levels, serving as a critical readout of neuronal stress signalling and the propagation of neuroinflammatory factors [44,45]. Neurotoxic stimuli and cell death are tightly coupled with mitochondrial stress and oxidative damage, ultimately compromising energy homeostasis within the CNS. Our data on triphosphates and the ATP/ADP ratio support PARP1 inhibition as beneficial, sustaining energetic homeostasis in chronic neuropathic pain. We then assessed the levels of critical amino acids involved in energy metabolism and neurotoxicity in the whole spinal cord content. We observed a significant increase in glycine following PARP1 inhibition, suggesting increased availability of this metabolite as a precursor of antioxidant molecules and protecting against oxidative stress [46]. Notably, glutamine and glutamate levels were also increased in olaparib-treated rats. These metabolites play multiple roles in supporting mitochondrial metabolism, specifically involving nitrogen transport, anaplerotic precursors for the tricarboxylic acid (TCA) cycle and glutathione synthesis [47]. Finally, PARP1 inhibition also increased GABA levels, further sustaining the TCA cycle and the osmoregulatory role of olaparib treatment [48].

To explore the relationships between the metabolic alterations and the molecular mechanisms underlying neuropathic pain and PARP1 inhibition, we performed an unbiased proteomic analysis. Our data revealed changes in the expression of a subset of proteins implicated in redox balance and neuroglial homeostasis. In particular, Gstm1, Gstt3, Ca1 and Prdx6 were found to be strongly increased in CCI rats and modulated by PARP1 inhibition. Gstm1 and Gstt3 are members of the glutathione S-transferase family and play pivotal roles in detoxifying ROS, counteracting oxidative damage [49]. Their functions have also been linked to neuroinflammation and intercellular communication between astrocytes and microglia, promoting glial crosstalk, particularly through the modulation of cytokine release between astrocytes and microglia [50]. The upregulation of Ca1, which is common in hypoxic conditions and acidic environments, may reflect a metabolic shift to sustain inflammatory stress and has previously been associated with maladaptive changes in neuropathic pain models [50]. Prdx6 is a key molecule expressed by astrocytes that functions as a redox sensor and mediator of neuroinflammatory signalling. Its overexpression has been linked to increased ROS production and prolonged astrocyte signalling, which in turn promotes reactive microgliosis [51]. Our data establish PARP1 as a master regulator of this set of proteins, damping redox-inflammatory coupling in the spinal cord.

To better clarify whether the observed metabolic and redox adaptation reflected cell-autonomous or hierarchical glial responses, we finally performed cell-type specific PARP1 KD in HMC3 and CCF-STTG1 co-cultures. Interestingly, selective PARP1 silencing in HMC3 cells (i.e. microglia) was sufficient to reproduce most of the metabolic adaptations observed following olaparib-mediated inhibition, including reduced PAR/pADPr accumulation, mitochondrial morphology remodelling, reduced basal OCR and mitochondrial ROS levels. Notably, these effects were not restricted to HMC3 cells, as PARP1 KD in microglia also affected mitochondrial parameters in co-cultured CCF-STTG1 cells. Conversely, CCF-STTG1-restricted PARP1 KD produced weaker effects on HMC3 cells, suggesting that PARP1 activity in microglia may represent an upstream component of the glial response to inflammatory priming.

Taken together, these findings delineate a multifaceted role for PARP1 in orchestrating the neuroinflammatory and metabolic rewiring that sustains chronic pain. By linking redox imbalance, glial plasticity and bioenergetic dysfunction, PARP1 emerges as a central node in the cascade triggered by nerve injury. Its hyperactivation drives PAR accumulation, amplifies oxidative stress and promotes reactive gliosis and cell death. In addition to confirm its enzymatic involvement, our study uncovers PARP1 as a regulatory protein that integrates inflammatory cues and metabolic adaptation. Olaparib not only mitigates the transition of neuropathic pain to a chronic state but also re-establishes the levels of proteins involved in redox balance, offering mechanistic insights into how PARP1 inhibition can reverse central sensitization and mitigate chronic pain states.

## 4 Materials and methods

**Cell cultures.** The experiments were performed on HMC3 (Cat.no. CRL-3304, RRID: CVCL_II76, ATCC) and CCF-STTG1 (Cat.no. CRL-1718, RRID: CVCL_1118, ATCC) human cell lines. HMC3 cells were cultured in growth medium consisting of Minimum Essential Medium (MEM, Cat.no. 32360026, Gibco) supplemented with 10% fetal bovine serum (FBS, Cat.no. 26140079, Gibco), l-glutamine 2 mmol/L (Cat.no. 25030024, Gibco) and 100 IU/mL penicillin-streptomycin solution (Pen-Strep, Cat.no. 15140-122, Gibco). CCF-STTG1 cells were cultured in growth medium consisting of RPMI-1640 (Cat.no. 11875-093, Gibco) and d(+)-glucose anhydrous 4.5 g/L (Cat.no. 1.02415, Merk) supplemented with 10% FBS, 1 mmol/L sodium pyruvate (Cat.no.11360-039, Gibco) and 100 IU/mL Pen-Strep. The cells were maintained under standard culture conditions, at 37 °C in a humidified atmosphere (95% air and 5% CO_2_) and were routinely subcultured in culture flasks.

HMC3 cells were transduced with the pCDH-CMV-MCS-EF1α-GFP-T2A-Puro lentiviral vector (Cat.no. CD513B-1, System Biosciences) to induce a stable expression of GFP. First, to obtain lentiviral particles, HEK293T cells were seeded at a final density of 50′000 cells per cm^2^ in Dulbecco's Modified Eagle Medium (DMEM, Cat. no. 10-013-CMR, Corning), supplemented with 10% FBS (Cat. no. 35-079-CV, Corning), 2 mmol/L of l-glutamine (Cat. no. 25-005-CI, Corning), and 100 UI/mL of penicillin-streptomycin solution (Cat. no. 30-001-CI, Corning) and incubated at 37 °C and 5% CO_2_. After 48 h, the culture medium was replaced, and the cells were transduced with the CD513B-1 lentiviral vector via Attractene transfection reagent (Cat.no. 301004, Qiagen) according to the manufacturer's instructions. Briefly, lentiviral packaging mix containing 2.19 μL of pRRE, 1 μL of pREV, 1.44 μL of pVSVg, 900 ng of plasmid DNA, 4.5 μL of attractene and 100 μL of Opti-MEM™ reduced serum medium (Cat. no. 31985062, Invitrogen) was added to the cells, which were then incubated at 37 °C and 5% CO_2_ overnight. The following day, the culture medium was replaced with complete DMEM without penicillin-streptomycin solution, and the HEK293T cells were maintained in the incubator for additional 30 h. Finally, the conditioned medium was collected and filtered through a 0.45 μm filter (Cat. no. SLHA033SS, Sigma-Aldrich) and stored at −80 °C until transduction. To induce the lentiviral transduction in HMC3 cells, cells were seeded at a final density of 30′000 cells per cm^2^. After 24 h, a lentiviral mixture containing 1 mL of filtered supernatant, 1 mL of complete growth medium and 8 μg/mL polybrene solution (Cat. no. H9268, Sigma Aldrich) was added to the cells. Spinfection was then performed via centrifugation at 1′800 rpm for 60 min at room temperature, after which the cells were incubated at 37 °C in 5% CO_2_. The next day, the culture medium was replaced, and transduced cells were selected for five days with 1.25 μg/mL of puromycin dihydrochloride (Cat.no. A1113803, Invitrogen). The efficiency of HMC3 transduction was assessed by immunocytochemistry.

For co-culture experiments, cells were seeded on 12 mm diameter coverslips in a 24-well plate. HCM3 GFP^+^ and CCF-STTG1 cells were co-seeded at a 1:1 ratio of 30′000 cells per cm^2^ per cell line to reach a final density of 60′000 cells per cm^2^. The co-culture medium consisted of HMC3 growth medium supplemented with the specific supplements required for CCF-STTG1 maintenance. After 24 h, the cells were stimulated with 50 ng/ml LPS (Cat.no. L2630, Sigma), a concentration used to selectively prime microglia (i.e., HMC3) and treated with 100 nM olaparib (Cat.no. S1060, Selleckcem) [52]. The cells were maintained for the next 4 h and then fixed with 4% paraformaldehyde (PFA, Cat.no. 16005, Sigma) with 2% (w/v) sucrose (Cat.no. 573113, Millipore) and incubated for 10 min at room temperature. The cells were then washed with phosphate-buffered saline (PBS) and stored at 4 °C until use.

**PARP1 knockdown (KD).** HMC3 and CCF-STTG1 cells were transiently transfected with PARP siRNA (ID: s1097, Life Technologies) or negative control siRNA (Cat.no. AM4611, Invitrogen) using Lipofectamine 3000 Transfection Reagent (Cat. no. L3000015, Invitrogen) according to the manufacturer's instructions. Briefly, 30′000 cells per cm^2^ were seeded under standard culture conditions. The following day, transfection was performed in serum-free medium for 6 h; then, the medium was replaced with complete growth medium. Cells were maintained in culture for 24 h (HMC3) or 48 h (CCF-STTG1) before the subsequent experiments.

**Immunocytochemistry**. For immunocytochemistry fixed cells were incubated with blocking solution consisting of 10% normal goat serum (NGS, Cat.no. ab7481, Abcam) and 0.1% Triton X100 (Cat.no. 282103, Sigma-Aldrich) in PBS, for 1 h at room temperature. Then, the cells were incubated overnight at 4 °C with the primary antibodies (1:200 anti-GFP antibody, Cat.no. ab13970, Abcam, RRID: AB_300798; 1:200 anti-Mitochondria antibody, Cat.no. ab92824, Abcam, RRID: AB_10562769; 1:100 anti-PAR/pADPr Antibody, Cat.no. 4335-MC-100, R&D System, RRID: AB_2572318), diluted in incubation solution (1% NGS in PBS-Triton X100 0.1%). On the following day, the coverslips were washed with 0.1% Triton X100 in PBS and incubated for 1 h at room temperature with an appropriate combination of the fluorescent secondary antibodies diluted in incubation solution (1:1′000, goat anti-chicken IgY H&L Alexa Fluor 488, Cat.no. ab150169, Abcam, RRID: AB_2636803; 1:1′000 goat anti-mouse Alexa Fluor 546, Cat.no. A-11003, Invitrogen, RRID: AB_2534071; 1:1′000 goat anti-mouse Alexa Fluor 647, Cat.no. A-21235, Invitrogen, RRID: AB_2535804). The samples were washed again with 0.1% Triton X100 in PBS, and the nuclei were counterstained with 4′,6-diamidino-2-phenylindole (DAPI, 1:1′000, Cat.no. D1306, Invitrogen) for 3 min at room temperature. Coverslips were then mounted with Fluoromount Aqueous Mounting Medium (Cat.no. F4680, Sigma-Aldrich). Digital images were acquired via a Leica TCS SP8 confocal microscope. The quantification of nuclear PAR/pADPr signals was performed via Fiji software (v. 2.16.0/1.54p). For each z-stack image, the nuclei were segmented via DAPI-positive staining, and the PAR/pADPr MFI was quantified. The PAR/pADPr MFI was normalized to the total DAPI-positive area. Mitochondrial morphology was analysed on mitochondria-stained z-stack images via the Mitochondrial Analyzer plugin for Fiji as previously described [53,54]. The quantitative parameters included the total branches length/mito and the number of individual mitochondrial particles.

**Extracellular flux (XF) analysis and mitochondrial stress test**. For XF assay, cells were seeded into Seahorse XFe24 cell culture microplates (Cat.no. 100777-004, Agilent) according to the co-culture protocol described above and cultured under standard conditions. The following day, cells were treated with 50 ng/ml LPS (Cat.no. L2630, Sigma) and/or 100 nM olaparib (Cat.no. S1060, Selleckchem) for 4 h. For PARP1 KD experiments, co-cultures were established using previously transfected HMC3 or CCF-STTG1 cells. After, culture medium was removed, cells were washed and supplemented Seahorse XF DMEM assay medium (Cat.no. 103575-100, Agilent) was added. Then, plates were incubated for 1h at 37 °C in a non-CO_2_ incubator for temperature and pH equilibration. Mitochondrial function was evaluated using the Seahorse XF Cell Mito Stress Test Kit (Cat.no. 103015-100, Agilent), as previously described [55]. Briefly, sequential injections of 1.5 μM oligomycin, 1 μM FCCP, and 0.5 μM rotenone/antimycin A were performed, and OCR and ECAR were measured using the Seahorse XFe24 Analyzer. Data were normalized to the total protein content per well and expressed as pmol/min/g for OCR and mpH/min/μg for ECAR.

**Flow cytometry**. For flow cytometry-assisted analysis, HMC3 and CCF-STTG1 cells were seeded at a final density of 40′000 cells/cm^2^ (1:1 ratio) in 6-well plates according to the protocol described above, and cultured for 12 h. For PARP1 KD experiments, co-cultures were established using previously transfected cells. To measure mitochondrial ROS levels, cells were stained with 2.5 μM of MitoSOX probe (Cat. no. M36008, Invitrogen) for 30 min at 37 °C, and then flow cytometry analysis was performed using the MACSQuant Analyser 16 (Miltenyi Biotec), as previously described [56]. Unstained CCF-STTG1 and HMC3-GFP^+^ cells were also analysed to establish the gating threshold for mitochondrial ROS quantification. Data were subsequently evaluated using FlowJo™ Software (BD Biosciences).

**Chronic constriction injury model and drug treatment**. N = 60 male Sprague-Dawley rats (6-10 weeks of age), were randomly assigned to different cages (n ≤ 3 animals per cage) and kept at a constant temperature (23-25 °C) with a 12:12 light/dark cycle and ad libitum standard laboratory diet and water.

Neuropathic pain was induced via the protocol first described by Bennet and Xie [57], with minor modifications [27]. On day 0, the rats were randomly divided into the 3 experimental groups and anaesthetized via isoflurane inhalation (i.e., 4% induction, 2% maintenance) following pre-emptive analgesia with meloxicam (5 mg/kg, intramuscularly). Throughout the surgical procedure, the animals were placed on a heated pad to maintain basic physiological parameters. The left hind paw was shaved, and the sciatic nerve was exposed via blunt dissection through the biceps femoris. For animals subjected to CCI procedures, the sciatic nerve was exposed and freed from any adhering tissues at the midthigh level, proximal to the trifurcation, and four ligatures (4/0 chromic silk, Ethicon) were tied around the nerve with ∼1 mm spacing between the sutures. Constriction of the nerve was minimal and immediately stopped if a brief twitch was observed to prevent the arrest of epineural blood flow. Finally, the wound was closed by suturing the skin (2/0 absorbable suture in PGA-PCL, Bioshort). Sham surgery was performed by exposing the sciatic nerve as described above without any ligatures. The rats were then transferred to their home cages and allowed to recover. From day 10 post-ligatures, when mechanical allodynia was fully established, until day 16, rats received a daily intraperitoneal (i.p.) injection of either vehicle (5% DMSO and 95% saline), or olaparib (5 mg/kg, Cat.no. HY-10162, MedChemExpress), depending on the group assignment. Injections were performed each morning at approximately 9:00 a.m., and behavioural tests were carried out approximately 2 h after injection, as described in the relevant sections.

**Von Frey test**. Mechanical allodynia was evaluated by measuring the paw withdrawal threshold in response to calibrated von Frey filaments. The rats were placed individually in an acrylic cage with a wire-grid floor for approximately 20 min to allow behavioural acclimation to the novel environment. Filaments ranging from 2 to 10 g were applied in ascending order to the plantar surface of the left hind paw following the up-and-down method [58]. Each filament was applied for 3-4 s, with a minimum interstimulus interval of 10 s. Paw withdrawal or licking in response to the stimulus was recorded after 3 trials. The data are presented as the mechanical withdrawal threshold in grams.

**Open field and open field grid walk tests**. OF and OFGW tests were performed at 0, 8 and 16 days post-ligatures (dpl). In the OF, the rats were individually placed in a 40 × 40 cm arena with a smooth floor and allowed to acclimate to the new experimental environment for 10 min to minimize stress. Locomotor activity was recorded for 3 min via a top-mounted camera. In the OFGW setting, the animals were placed in an identical arena fitted with a 2 cm^2^ mesh floor. A dual-camera system was used: the top-mounted camera recorded the overall distance covered, whereas a lateral camera recorded footfall errors. Videos were analysed offline via Ctrax (v0.5.18, Caltech).

**Kinematic analysis.** For kinematic analysis, at 16 dpl n = 3 rats per group (sham vehicle, CCI vehicle, and CCI olaparib) were placed in a clear Plexigals runway, 40 cm long, 10 cm wide and 15 cm high to assess over ground locomotion. Anatomical landmarks on the hindlimb were highlighted at the iliac crest, hip joint, knee joint, ankle and toe. For each rat in each group, 6-10 continuous gait cycles were captured using a recording camera. The kinematic data were analysed by an investigator blinded to the treatment group using Tracker software (v 6.3.4) to obtain knee angle, ankle angle, stride duration, stride length, walking speed, swing phase, stance phase, and duty factor.

***Ex vivo* tissue pathology.** The day of sacrifice, the rats were deeply anaesthetized via i.p. injection of ketamine (10 mg/mL) combined with xylazine (1.17 mg/mL), and subsequently transcardially perfused with saline, followed by fixation with ice-cold 4% PFA in PBS. Spinal cords were isolated and post-fixed in 4% PFA in PBS at 4 °C overnight. On the following day, the tissues were washed in PBS and left for at least 48-72 h in 30% (w/v) sucrose (Cat.no. 573113, Millipore) in PBS for cryoprotection. The spinal cords were then embedded in optimum cutting temperature (OCT) medium (Cat.no. 0782, Kaltek) and snap frozen in liquid nitrogen. 20 μm thick slices were cryosectioned via a cryostat and mounted on Superfrost Plus microscope slides (Cat.no. 10149870, Epredia), then stored at – 80 °C until use.

For quantification of glial markers and PAR polymers accumulation, the sections were washed in PBS and pre-treated with 3% H_2_O_2_ in PBS for 15 min at room temperature. The slides were then incubated and permeabilized in the blocking solution (10% NGS, 0.3% Triton X100 in PBS) for 1 h at room temperature. Primary antibodies (1:100 mouse anti-GFAP antibody, Cat.no. 620566, BD transduction Laboratories, RRID: AB_397916; 1:100 goat anti-AIF/Iba1 antibody Cat.no. NB100-1028, Novus Biologicals, RRID: AB_521594; 1:100 mouse anti-PAR/pADPr antibody, Cat.no. 4335-MC-100, R&D System, RRID: AB_2572318), were diluted in 0.3% Triton X100 in PBS and incubated for 1 h at room temperature. Then, the samples were incubated with a biotinylated secondary antibody (1:200 horse anti-mouse/rabbit/goat IgG Antibody (H + L), Cat.no.BA-1300, Vector Laboratories, RRID: AB_233618), diluted in 0.3% Triton X100 in PBS containing 1% of bovine serum albumin (Cat.no. A2058, Sigma-Aldrich) for 30 min. After washing, the slides were incubated with avidin-biotin complex-horseradish peroxidase (ABC-HRP, Cat.no. PK-7100, Vector Laboratories) for 30 min at room temperature. The samples were then washed in 0.3% Triton X100 in PBS and exposed to a solution of 1% 3,3′-diaminobenzidine (DAB) supplemented with 0.3% H_2_O_2_ in PBS until a brown colouration appeared. The reaction was blocked by dipping the sections into distilled water, and the sections were counterstained with hematoxylin (Cat.no. MHS32, Sigma-Aldrich). The sections were then dehydrated with increasing alcohol solutions, cleared with xylene and coverslipped with synthetic mounting medium (Eukitt Quick-hardening mounting medium, Cat.no. 03989, Sigma-Aldrich). Digital images were acquired via a Nexcope NIB600 biological microscope. The percentage of the area positive for each marker was quantified via Fiji software. Brightfield images were converted to 8-bit grayscale. A consistent threshold was applied to segment the positive signal for each round of staining on the basis of colour deconvolution, and the percentage of stained area within the ipsilateral dorsal horn was calculated.

To evaluate motoneuronal integrity, spinal cord sections were processed for Nissl staining as previously described [59]. Briefly, sections were dehydrated through increasing ethanol concentrations, cleared in xylene, and rehydrated through decreasing ethanol concentrations. Sections were then incubated with cresyl violet (Cat.no. C5042, Sigma-Aldrich) for 10 min, rinsed in deionized water, dehydrated through graded ethanol solutions, cleared with xylene and coverslipped. Digital images were acquired via a Nexcope NIB600 biological microscope.

For the quantification of apoptotic and DNA damage markers and Cx43 expression in glial cells, spinal cord sections were rinsed with PBS, and then blocked for 1 h at room temperature in blocking buffer consisting of 10% NGS or Normal Donkey Serum (NDS, Cat.no. ab7475, Abcam, RRID: AB_2885042) in 0.3% Triton X100 in PBS. Then, the sections were incubated overnight at 4 °C with the primary antibodies (1:100 mouse anti-GFAP antibody, Cat.no. 620566, BD transduction Laboratories, RRID: AB_397916; 1:100 goat anti-AIF/Iba1 antibody, Cat.no. NB100-1028, Novus Biologicals, RRID: AB_521594; 1:100 anti-NeuN antibody, Cat.no. ab104224, Abcam, RRID: AB_10711040; 1:100 Histone H2A.X polyclonal antibody, Cat.no. PA5-28778, Invitrogen, RRID: AB_2546254, 1:100 anti-Cx43 antibody, Cat.no. 3512, Cell Signalling, RRID: AB_2294590; 1:100 Anti-YKL-40/CHI3L1 antibody, Cat.no. ab77528, Abcam, RRID: AB_2040911) diluted in 1% NGS or NDS, and 0.3% Triton X100 in PBS. The following day, the slides were washed in 0.3% Triton X100 in PBS and incubated with the appropriate secondary antibodies (1:1′000 goat anti-mouse Alexa Fluor 488, Cat.no. A11001, Invitrogen, RRID: AB_2534069; 1:1′000 donkey anti-goat Alexa Fluor 546, Cat.no. A11056, Invitrogen, RRID: AB_2534103; 1:1′000 goat anti-mouse Alexa Fluor 546, Cat.no. A11003, Invitrogen, RRID: AB_141370; 1:1′000 goat anti-rabbit Alexa Fluor 647, Cat. no. A21244, Invitrogen, RRID: AB_2535812) diluted in 1% NGS or NDS, and 0.3% Triton X100 in PBS for 1 h at room temperature. After washing in PBS, the nuclei were counterstained with DAPI (1:1′000, Cat.no. D1306, Invitrogen) for 3 min and then mounted with Fluoromount Aqueous Mounting Medium (Cat.no. F4680, Sigma-Aldrich). Digital images were acquired via a Leica TCS SP8 confocal microscope. Double-positive cells for NeuN/Cl Casp3, Gfap/Cl Casp3, Iba1/Cl Casp3, and H2A-X/Dapi were counted in randomized regions of interest (ROIs) and expressed as the number of cells per mm^2^. The fluorescence intensities of Gfap, Iba1 and Cx43 were analysed via Fiji software. For each population marker, a profile plot was generated and superimposed on the corresponding Cx43 fluorescence profile plot.

Microglial morphological parameters were obtained from Iba1-stained z-stack images converted to 8-bit grayscale. A consistent threshold was applied to segment the positive signal for each cell on the basis of color deconvolution, and Fiji software plugins for quantification of area, perimeter and circularity were used. A total number of n ≥ 6 cells per ROI were calculated, and the average area, perimeter and circularity were obtained from n = 3 rats per group. Colocalization between Chi3L1 and Gfap marker was analysed using the Coloc 2 plugin in Fiji software. Pearson's correlation coefficient was calculated from dual-channel confocal immunofluorescence images on spinal cord sections from n = 3 rats per group.

**Immunoblot**. For the quantification of IL-1β expression, HMC3 cells were seeded in a standard culture flask at a final density of 40′000 cells per cm^2^. The following day, the cells were treated with vehicle (i.e., PBS+1% DMSO), LPS + vehicle (Cat.no. L2630, Sigma), olaparib + vehicle (Cat.no. S1060, Selleckcem), and LPS + olaparib and cultured for 4 h. The cells were then collected, and proteins were extracted via RIPA lysis buffer (100 μL/sample; Cat.no. ab156034, Abcam), supplemented with protease inhibitor (1:100, Cat.no. P8340, Merck). For the validation of PARP1-KD, protein extracts were collected from previously transfected HMC3 and CCF-STTG1 cells. An equal amount of protein extract (10 μg) was electrophoresed on 4-15% Mini-PROTEAN TGX gels (Cat.no. 4561083, Bio-Rad) and then transferred to nitrocellulose membranes via a Trans-Blot Turbo Transfer System (Cat.no. 1704150, Bio-Rad). The membranes were incubated for 1 h at room temperature with blocking buffer consisting of 5% non-fat milk (Cat. no. A0830,0500; PanReac AppliChem), in 0.1% Tween 20 (Cat.no. A4974,0500, PanReac AppliChem) in PBS and then incubated overnight at 4 °C with anti-IL-1β antibody (1:500, Cat.no. A278004, Antibodies, RRID: n.a.), Anti-PARP1 antibody (1:1′000, Cat.no. ab227244, Abcam, AB_3676687) and anti-Gapdh (1:10′000, Cat.no. ab181602, Abcam, RRID: AB_2630358) diluted in blocking buffer. The following day, the membranes were washed with 0.1% Tween 20 in PBS and then incubated for 1 h at room temperature with goat anti-rabbit IgG (H + L) secondary antibody, HRP (1:10′000, Cat.no. 31460, Invitrogen, RRID: AB_228341), diluted in blocking buffer.

For the quantification of arginase 1 and PAR/pADPr expression, spinal cord proteins were extracted from the rats and snap frozen in liquid nitrogen. The samples were then mechanically triturated in a solution of RIPA lysis buffer supplemented with protease inhibitor (10 μL/mg) and incubated at 4 °C for 20 min. The samples were subsequently centrifuged at 13′000 g for 10 min at 4 °C. The supernatants were collected, and 10 μg of protein extract was electrophoresed on 4-15% Mini-PROTEAN TGX gels (Cat.no. 4561084, Bio-Rad) for each sample. The proteins were transferred to nitrocellulose membranes (Cat.no. 1704158, Bio-Rad), using a Trans-Blot Turbo Transfer System. The membranes were incubated for 1 h at room temperature with blocking buffer consisting of 5% non-fat milk in 0.1% Tween 20 in PBS. Afterwards, the membranes were incubated with an anti-arginase 1 antibody (1:1′000, Cat.no. sc-166920, Santa Cruz Biotechnology, RRID: AB_10609486), anti-PAR/pADPr antibody (1:1′000, Cat.no. 4335-MC-100, R&D System, RRID: AB_2572318) and anti-Gapdh antibody (1:1′000, Cat.no. ab181602, Abcam, RRID: AB_2630358) diluted in blocking buffer. The following day, the membranes were washed with 0.1% Tween 20 in PBS and incubated for 1 h at room temperature with a goat anti-rabbit IgG (H + L) secondary antibody, HRP (1:10′000, Cat.no. 31460, Invitrogen, AB_228341) or a goat anti-mouse IgG (H + L) secondary antibody, HRP (1:5′000, Cat.no. 31430, Invitrogen, RRID: AB_228307), diluted in blocking buffer. The protein bands were detected via a ChemiDoc system (Cat.no. 12003153, Bio-Rad). The density of each band was quantified via Fiji software, and the band density was normalized to the loaded control optical density measured in the same membrane. Uncropped membranes are shown in **Supplementary information file – Uncropped Gels and Blots**.

**Sample preparation for MS-based proteomic analysis**. Spinal cords were isolated from the rats and snap frozen in liquid nitrogen. The samples were then mechanically triturated in a solution of RIPA lysis buffer supplemented with protease inhibitor (10 μL/mg) and incubated at 4 °C for 20 min. The samples were subsequently centrifuged at 13′000 g for 10 min at 4 °C. The supernatants, which contained protein extracts, were collected and stored at −80 °C. Prechilled acetone (−20 °C) was used to precipitate the protein content of all the samples. After centrifugation at 10′000 g, the pellet was resuspended in 8 M urea, and the total amount of protein was quantified via the BCA protein assay kit (Cat.no. J63283.QA, Thermo Fisher Scientific). The reduction and alkylation of the protein sulfhydryl groups were carried out via 1.6 mM dithiothreitol and 7 mM iodoacetamide, respectively. The diluted protein samples (1:10 with 50 mM NH_4_HCO_3_) were treated with trypsin at 37 °C for 16 h (enzyme-to-protein ratio of 1:50 w/w). The final samples were analysed via Liquid Chromatography-Mass spectrometry (LC-MS).

**Analysis of tryptic digests by LC-MS**. Hydrolytic peptides were separated and detected via a UHPLC system (Thermo Fisher Scientific Dionex UltiMate 3000 RSLCnano) coupled with an Orbitrap Fusion Tribrid mass spectrometer (Q-OT-qIT, Thermo Fisher Scientific). The samples were diluted with a 5% aqueous formic acid (FA) solution. Peptides were eluted on a PepMap® RSLC C18 column (EASYSpray, 75 μm × 50 cm, 2 μm, 100 Å, Thermo Fisher Scientific) and separated by elution at a flow rate of 0.250 μL/min at 40 °C, with a linear gradient of solvent B (CH_3_CN + 0.1% FA) in solvent A (H_2_O + 0.1% FA). The eluted peptides were ionized via a nanospray (Easy-spray ion source, Thermo Scientific) using a capillary temperature and voltage set to 275 °C and 2.0 kV, respectively. Peptide precursor scans in the *m*/*z* range of 400-1600 were performed with a resolution of 120′000 (200 *m*/*z*) with an AGC target for Orbitrap detection of 4.0 × 10^5^ and a maximum injection time of 50 ms. MS/MS spectra were acquired using a normalized collision energy (HCD) of 35. The dynamic exclusion duration was set to 60 s with a tolerance of 10 ppm around the selected precursor and its isotopes. The AGC target values and maximum injection times (ms) for the MS/MS spectra were 1.0 × 10^4^ and 100, respectively. The mass spectrometer was calibrated with Pierce® LTQ Velos ESI positive ion calibration solution (Thermo Fisher Scientific). MS data acquisition was performed via Xcalibur software v. 3.0.63 (Thermo Fisher Scientific). The mass spectra were analysed via MaxQuant software (version 2.5.1.0). The initial maximum allowed mass deviation was set to 6 ppm for monoisotopic precursor ions and 0.5 Da for MS/MS peaks. The enzyme specificity was set to trypsin, defined as the C-terminus to arginine and lysine, excluding proline, with a maximum of 2 missed cleavages allowed. Carbamidomethylcysteine was set as a fixed modification, whereas N-terminal acetylation and methionine oxidation were set as variable modifications. The spectra were analysed via the Andromeda search engine against the *Rattus Norvegicus* Uniprot sequence database. Quantification in MaxQuant was performed via the integrated Label-Free Quantification (LFQ) algorithm, which is based on extracted ion chromatograms (XIC) and employs fast LFQ. The false discovery rate was set to 1% at the peptide level and 1% at the protein level, with a minimum peptide length of seven amino acids required. Only proteins that were present and quantified in at least 2 out of 3 technical replicates were considered positively identified in a sample and used for statistical analyses.

The interaction networks of the significantly modulated proteins in clusters 1-4 were constructed via the String (https://string-db.org/) online tool for gene ontology. The minimum required interaction score was 0.150, with a maximum number of interactors of the first and second shells of interaction ≤5. Network interaction thickness indicates the strength of data support expressed as confidence of the textmining, experiments, databases, co-expression, neighbourhood, gene fusion, and co-occurrence. The k-means clustering method was used for the String network. Functional enrichment visualization was obtained via the String gene ontology tool. For functional enrichment analysis, terms were grouped by similarity ≥0.8, sorted by signal, and the number of genes was expressed as a circle dimension.

**Three-dimensional uniform manifold approximation and projection (UMAP)**. Analysis was performed via RStudio software (v2023.12.1 + 402) running the *umap*, *ggplot2*, *ggforce* and *dplyr* packages. UMAP was applied via default parameters with the number of output dimensions set to 3 (i.e., UMAP1-3). The resulting 3D embedding was combined with sample metadata for visualization. The group identities were mapped to colour, and the plots were rendered via *ggplot2* for a clear representation of spatial separation across the experimental conditions.

**HPLC analysis of metabolites**. After sacrifice, the spinal cords were isolated from the rats and snap frozen in liquid nitrogen. Then, the tissue samples were weighed and subjected to deproteinization by homogenization in a ratio of 1:10 with a precipitating solution composed of ice-cold HPLC-grade acetonitrile (Cat.no. 20060320, Merck), mixed with 10 mM potassium dihydrogen phosphate (pH 7.4, Cat.no. P0662, Merck) at a 3:1 (v/v) ratio. Homogenization was performed via an Ultra-Turrax disperser (Janke & Kunkel, Staufen, Germany) operating at 24′000 g for 90 s. The homogenates were then centrifuged at 20′690 g for 10 min at 4 °C. The resulting supernatants were extracted by adding HPLC-grade chloroform, followed by vigorous mixing and centrifugation at maximum speed for 5 min. The upper aqueous phase was carefully collected and used for metabolite analysis. Separation and quantification of energy phosphate compounds, oxidized/reduced forms of nicotinamide cofactors, purines, pyrimidines, antioxidants, and markers of oxidative/nitrosative stress were performed as previously described [60]. Amino acids and other amine-containing compounds were separated and quantified according to chromatographic conditions previously set up in our laboratory. In both cases, metabolites were analysed via a high-performance liquid chromatography (HPLC) system composed of a Spectra System P4000 pump coupled to a Hypersil C180 column (250 × 4.6 mm, 5 μm particle size) equipped with a matching guard column. Detection was achieved via a UV6000 LP diode array detector with a 5 cm light-path flow cell (Thermo Fisher Scientific). Overall, the 2 analyses allowed measurement of the concentrations of 57 low-molecular-weight, water-soluble metabolites in tissue extracts on the basis of comparisons with ultrapure external standards of known concentrations.

Metabolomic data were analysed via MetaboAnalyst 5.0. For functional pathway analysis, the complete set of identified metabolites across all experimental groups was loaded, and the KEGG pathway library for *Rattus norvegicus* was used. Enrichment analyses of the biological pathways in the sham vehicle vs. CCI vehicle, CCI vehicle vs. CCI olaparib, and sham vehicle vs. CCI olaparib groups was performed using metabolite abundance with no additional normalization, transformation or scaling. Samples were assigned to each experimental group, and metabolite names were mapped to database identifiers. Pathway analysis was performed using KEGG pathway mapping and results were visualized as biplot of pathway impact and –log_10_(p-value).

**Principal component analysis (PCA)**. To calculate the composite score of behavioural analysis and functional outcomes, the following parameters were considered as variables and loaded in the PCA: swing phase, stance phase, duty factor, OF distance, OFGWT distance, no. of footfalls, stride duration, stride length, walking speed, and withdrawal threshold. The percentage of variance explained by each PC was calculated and expressed as percentage contribution to PCs. For PCA on metabolites, metabolite abundance was used to calculate PCs and the percentage of variance explained by each PC. The PCA results were visualized as biplots displaying both sample distribution and variable loading, with directional color-coded vectors indicating individual metabolite contributions. All analyses and visualizations were performed via RStudio software, with the *FactoMineR*, *factoextra* and *corrplot* packages.

**Statistical analyses**. Data analysis was performed via GraphPad Prism software version 8.0.1. All the data are shown as the means ± SDs, and the sample sizes for each experiment are indicated in the figure legends. All the quantifications were performed by investigators blinded to the experimental conditions. No animals or data points were excluded *a priori*. Outlier detection was carried out via the ROUT method (Q = 1%). Data distributions were assessed for normality via either the Shapiro-Wilk test or the D'Agostino-Pearson omnibus test, depending on the sample size. Homogeneity of variance was verified prior to group comparisons. For datasets that met both criteria, comparisons between groups were evaluated via the appropriate test, reported in the figure legends. Non-normally distributed datasets were analysed via the Kruskal-Wallis test. A p-value <0.05 was considered statistically significant for all comparisons.

## Ethics approval

All experiments were performed in accordance with the principles of the Basel Declaration as well as with European and Italian regulations (2010/63/EU and Italian D. Lgs. no. 26/2014). All animal procedures were approved by the local committee for animal welfare (OPBA, University of Catania, Catania, Italy) and by the Italian Ministry of Health.

## Availability of data and materials

The datasets supporting the conclusions of this article are included within the article and its additional files.

## Declaration of generative AI and AI-assisted technologies in the manuscript preparation process

During the preparation of this work the authors used OpenAI in order to improve language and readability. After using this tool, the authors reviewed and edited the content as needed and take full responsibility for the content of the published article.

## CRediT authorship contribution statement

**Simona Denaro:** Conceptualization, Data curation, Formal analysis, Investigation, Methodology, Project administration, Resources, Writing – original draft, Writing – review & editing. **Simona D'Aprile:** Formal analysis, Investigation, Methodology, Writing – review & editing. **Anna Gervasi:** Formal analysis, Investigation, Methodology, Writing – review & editing. **Vincenzo Russo:** Formal analysis, Investigation, Methodology, Writing – review & editing. **Francesco Bellia:** Formal analysis, Investigation, Methodology, Writing – review & editing. **Sebastiano Giallongo:** Investigation, Methodology, Writing – review & editing. **Alessandro Lavoro:** Investigation, Methodology, Writing – review & editing. **Saverio Candido:** Investigation, Methodology, Writing – review & editing. **Alice Braga:** Formal analysis, Writing – review & editing. **Alexander V. Gourine:** Formal analysis, Writing – review & editing. **Giovanni Li Volti:** Resources, Writing – review & editing. **Lorella Pasquinucci:** Resources, Writing – review & editing. **Angela Maria Amorini:** Formal analysis, Investigation, Methodology, Writing – review & editing. **Carmela Parenti:** Investigation, Methodology, Resources, Writing – review & editing. **Rosalba Parenti:** Conceptualization, Data curation, Formal analysis, Project administration, Resources, Writing – original draft, Writing – review & editing. **Nunzio Vicario:** Conceptualization, Data curation, Formal analysis, Investigation, Methodology, Project administration, Resources, Writing – original draft, Writing – review & editing.

## Declaration of competing interest

The authors declare that they have no known competing financial interests or personal relationships that could have appeared to influence the work reported in this paper.
