# Supplementary material for: PARP1 rewires neuroinflammatory and redox metabolism associated with reactive neuroglia in neuropathic pain
Source: Redox Biol. 2026 Jun 18;95:104268. doi: 10.1016/j.redox.2026.104268 (PMC13312493; doi:10.1016/j.redox.2026.104268)
Supplement: Multimedia component 1 [file mmc1.pdf]

Supplementary information for

PARP1 rewires neuroinflammatory and redox metabolism associated with reactive neuroglia in neuropathic pain

Simona Denaro[1], Simona D'Aprile[2], Anna Gervasi[1], Vincenzo Russo[1], Francesco Bellia[3], Sebastiano Giallongo[2], Alessandro Lavoro[4], Saverio Candido[4], Alice Braga[5], Alexander V. Gourine[5], Giovanni Li Volti[3], Lorella Pasquinucci[6], Angela Maria Amorini[3], Carmela Parenti[7], Rosalba Parenti[1,*], Nunzio Vicario[1,*].

[1]Section of Physiology, Department of Biomedical and Biotechnological Sciences, University of Catania, 95123 Catania, Italy.

[2]Department of Medicine and Surgery, University of Enna "Kore", 94100 Enna, Italy.

[3]Section of Biochemistry, Department of Biomedical and Biotechnological Sciences, University of Catania, 95123 Catania, Italy.

[4]Section of General Pathology, Department of Biomedical and Biotechnological Sciences, University of Catania, 95123 Catania, Italy.

[5]Centre for Cardiovascular and Metabolic Neuroscience, Neuroscience, Physiology and Pharmacology, University College London, WC1E 6BT London, United Kingdom.

[6]Section of Medicinal Chemistry, Department of Drug and Health Sciences, University of Catania, 95123 Catania, Italy.

[7]Section of Pharmacology and Toxicology, Department of Drug and Health Sciences, University of Catania, 95123 Catania, Italy.

***Corresponding authors:**

Rosalba Parenti (email: parenti@unict.it);

Nunzio Vicario (email: nunziovicario@unict.it).

Keywords: Reactive gliosis, central sensitization, PAR polymers, olaparib, glutathione.

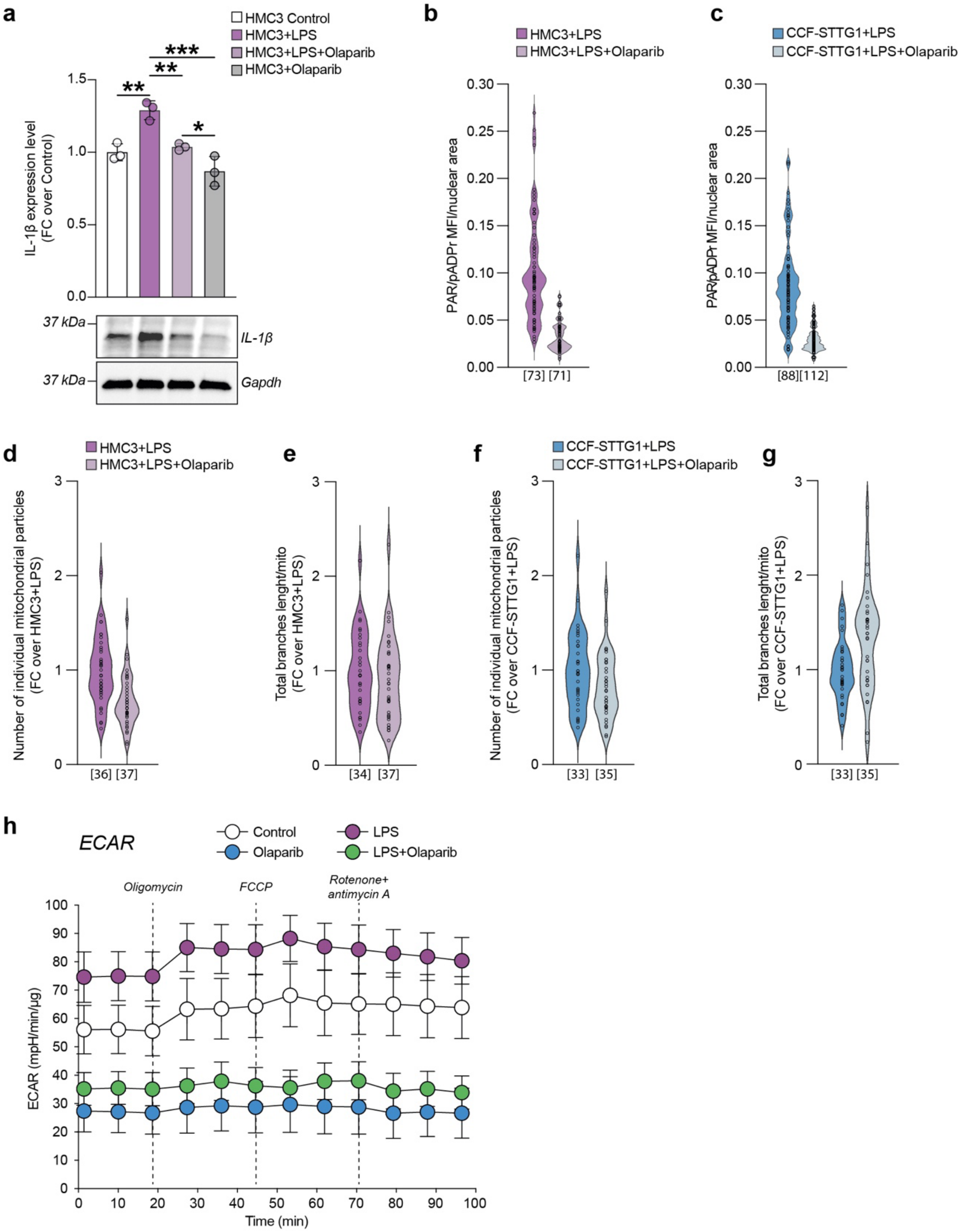

Fig. S1. IL-1β expression levels and single-cell analyses of the effects of PARP1 inhibition on glial cells following inflammatory stimulation. (a) Quantification and representative blot of the relative expression level of IL-1β in HMC3 control, HMC3+LPS, HMC3+LPS+Olaparib and HMC3+Olaparib. The data are reported as dot plots and means ± SDs of n = 3 biological replicates. *p-value < 0.05; **p-value < 0.01; ***p-value < 0.001, one-way ANOVA followed by the Holm-Šídák multiple-comparison test. (b) Single-cell quantification of the MFI of nuclear PAR/pADPr in HMC3+LPS and HMC3+LPS+Olaparib, related to

Figure 1c. (c) Single-cell quantification of the MFI of nuclear PAR/pADPr in CCF-STTG1+LPS and CCF-STTG1+LPS+Olaparib, related to Figure 1d. (d-e) Single-cell quantification of the number of individual mitochondrial particles and total branches length/mito in HMC3+LPS and HMC3+LPS+Olaparib, related to Figure 1f and 1g. (f-g) Single-cell quantification of the number of individual mitochondrial particles and total branches length/mito in CCF-STTG1+LPS and CCF-STGG1+LPS+Olaparib, related to figure 1h and 1i. The data are reported as aligned dot plots and violin plots. The number of analysed cells is shown on the x-axis. (h) Normalised ECAR of control, LPS, olaparib, and LPS+olaparib HMC3 and CCF-STTG1 co-cultures at 4 hrs, during sequential injections of 1.5 μM oligomycin, 1 μM FCCP, and 0.5 μM rotenone/antimycin A. Data are expressed as mean ± SEM of $n \geq 4$ biological replicates. ECAR: extracellular acidification rate, FC: fold change, LPS: lipopolysaccharide, MFI: mean fluorescence intensity.

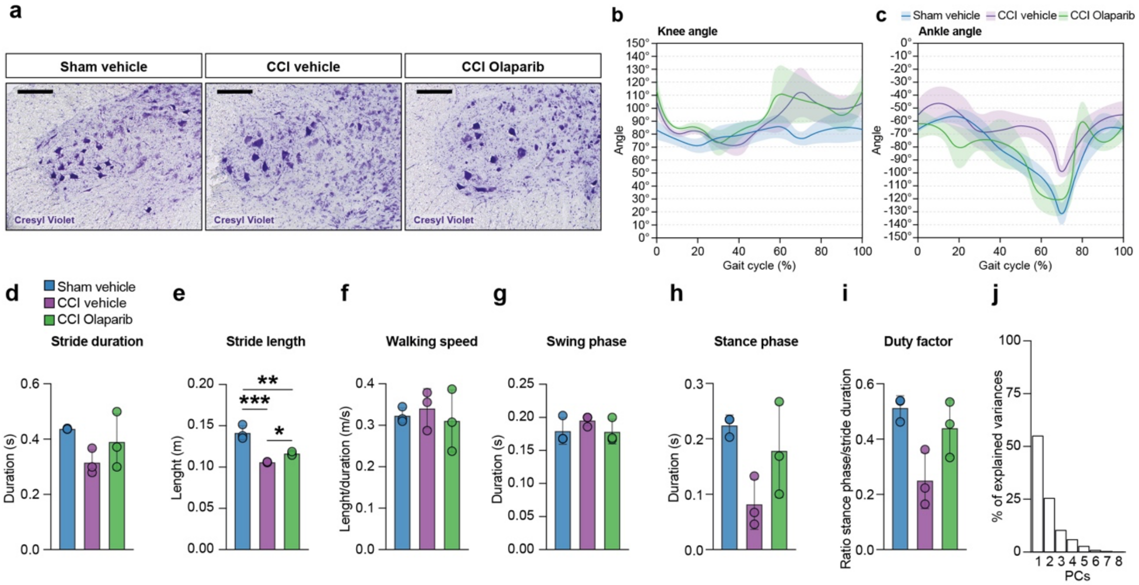

Fig. S2. Nissl-staining of ventral motor horns of the spinal cord and kinematic analysis of motor performance of sham vehicle, CCI vehicle, and CCI olaparib rats. (a) Analysis of Nissl-stained spinal cord samples of sham vehicle, CCI vehicle, and CCI olaparib rats. Scale bar = 200 µm. (b-c) Left hindlimb knee (b) and ankle (c) angle oscillation trace during gait cycle of sham vehicle, CCI vehicle, and CCI olaparib rats. (d-i) Kinematic analysis of the gait parameters stride duration (d), stride length (e), walking speed (f), swing phase (g), stance phase (h), and duty factor (i) in sham vehicle, CCI vehicle, and CCI olaparib rats. The data are shown as dot plots and means ± SDs of n ≥ 5 biological replicates, and *p-value < 0.05, **p-value < 0.01, ***p-value < 0.001; one-way ANOVA followed by the Holm-Šídák multiple-comparison test. (j) Scree plot showing the percentage of total variance explained by each PC of the PCA in figure 2k-l. PC: principal component, PCA: principal component analysis.

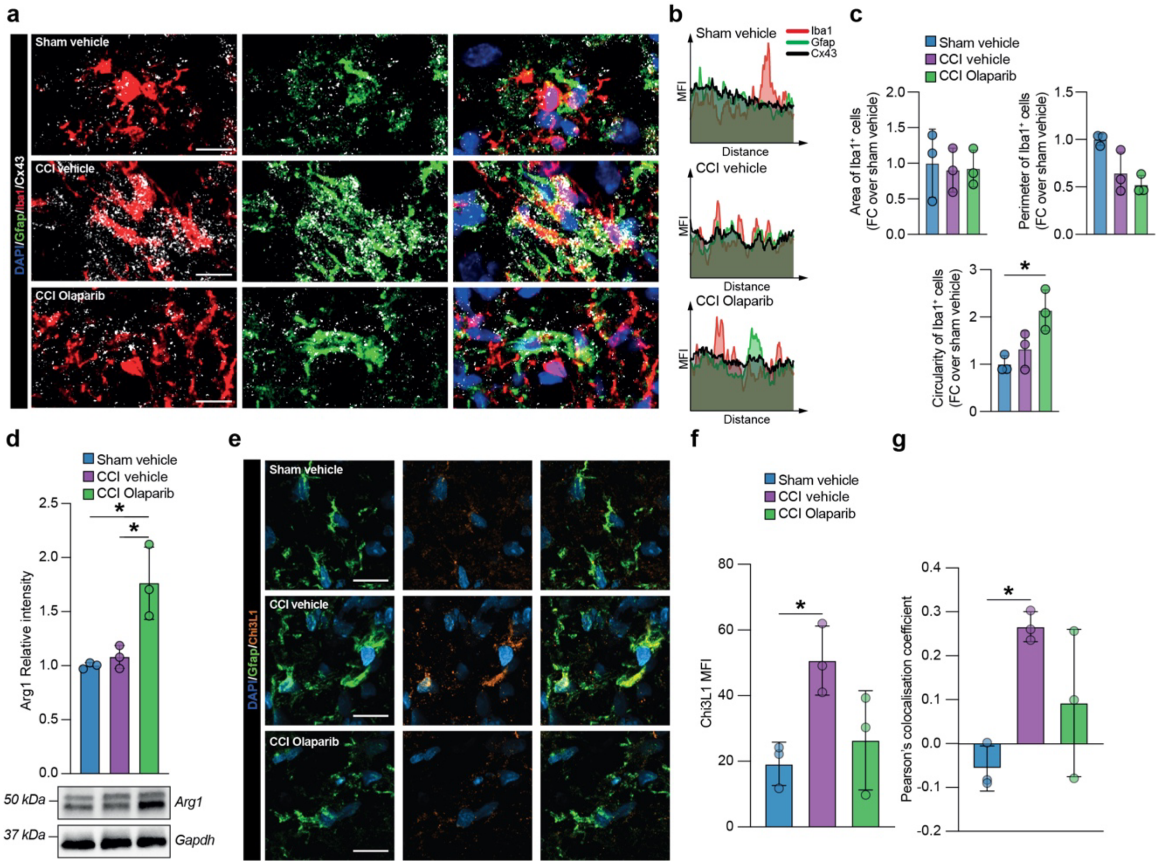

Fig. S3. Cx43-mediated astrocyte-microglia coupling, microglia morphology, Arg1 expression and spinal Chi3L1 staining. (a) Representative images of Iba1 (red), Gfap (green) and Cx43 (white) expression in the sham vehicle, CCI vehicle and CCI olaparib groups. Scale bars = 10 µm. (b) Peak profile plot of Iba1, Gfap and Cx43 MFI across groups. (c) Quantification of Iba1-positive cell morphological parameters: area, perimeter and circularity, in the sham vehicle, CCI vehicle and CCI olaparib groups. (d) Quantification and representative blot of the relative intensity of Arg1 in the sham vehicle, CCI vehicle and CCI olaparib groups. (e-g) Representative images of Chi3L1 (orange) and Gfap (green) expression (e), quantification of Chi3L1 MFI, and the Pearson's colocalization coefficient between Chi3L1 and Gfap (f) in the sham vehicle, CCI vehicle and CCI olaparib spinal cord dorsal horns. Scale bars = 10 µm. The data are reported as dot plots and means ± SDs of n = 3 rats per group. *p-value < 0.05; one-way ANOVA followed by the Holm-Šídák multiple-comparison test. FC: fold change, MFI: mean fluorescence intensity.

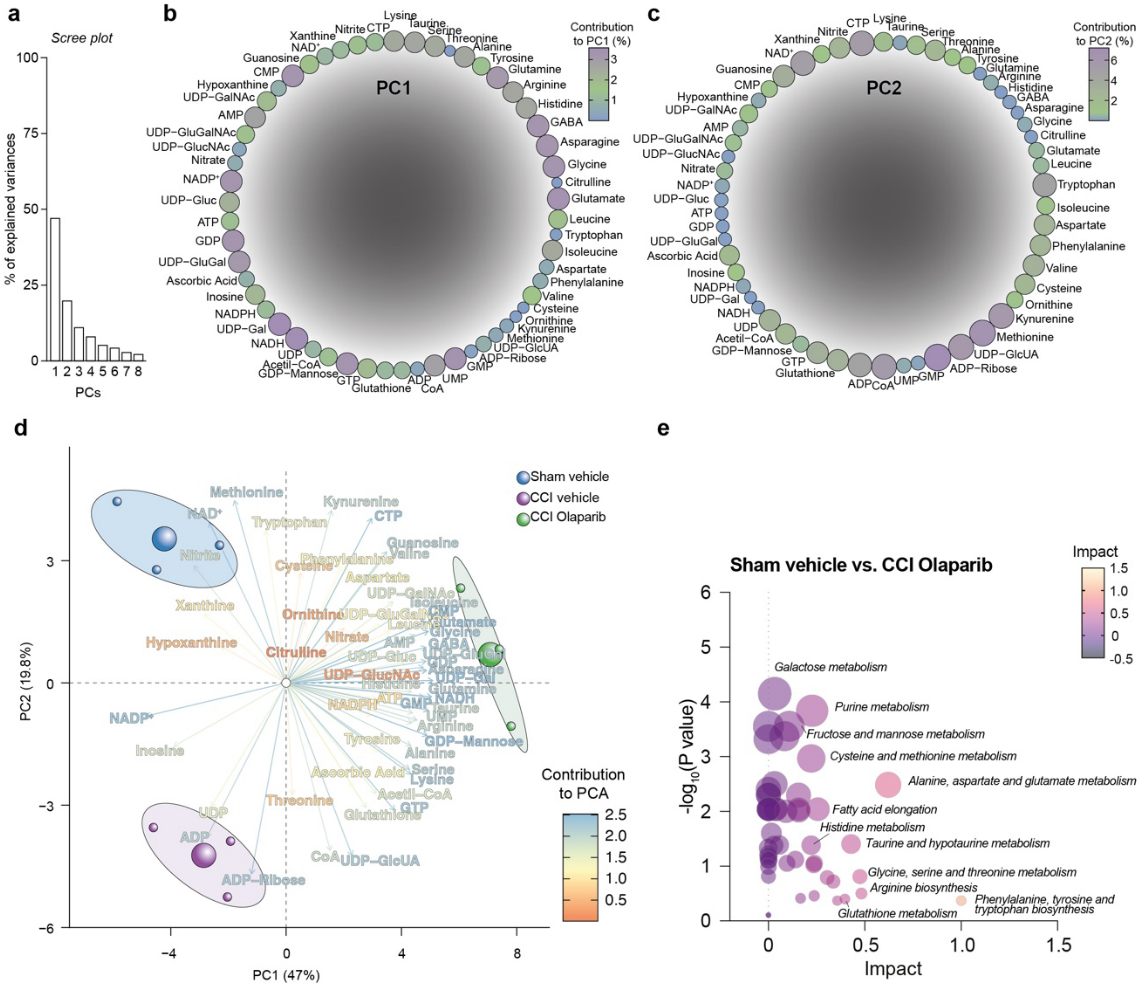

Fig. S4. Principal component analysis (PCA) and enrichment analysis of spinal cord metabolites. (a) Scree plot showing the percentage of total variance explained by each PC of the PCA. (b-c) Random circular bubble plot of the relative contribution of measured variables to PC1 (b) and PC2 (c) derived from PCA. Circle size is proportional to the contribution of the variable to PC1 or PC2, and colour intensity indicates the contribution as percentage. (d) PCA biplot for the sham vehicle, CCI vehicle and CCI olaparib groups. The key-colored arrows represent the contribution of each variable to the PCA. The small dots represent each single biological replicate, and the large dots represent the mean points of the confidence ellipses. (d) Data enrichment analyses of statistically and biologically relevant pathways in the sham vehicle vs. CCI olaparib groups. PC: principal component, PCA: principal component analysis.

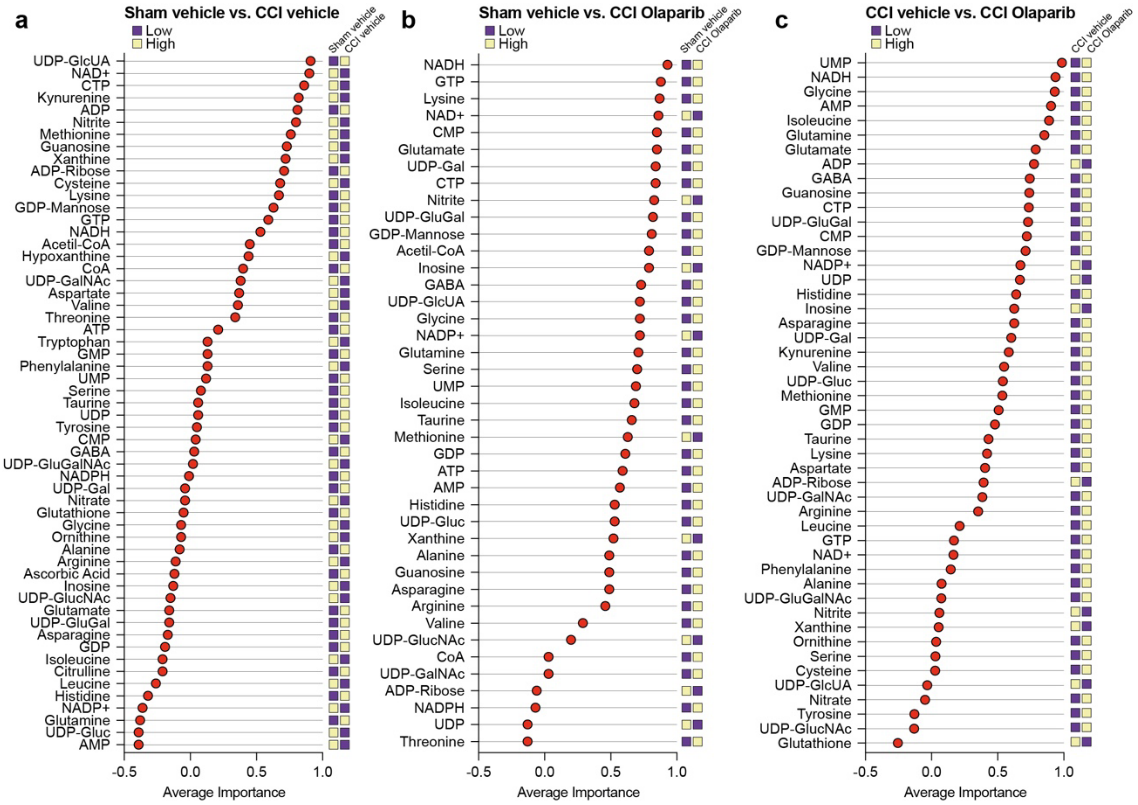

Fig. S5. Average importance analysis of metabolites in the indicated pairwise comparisons. (a-c) Analysis of the average importance of the tested metabolites between sham vehicle vs. CCI vehicle (a), sham vehicle vs. CCI Olaparib (b), and CCI vehicle vs. CCI Olaparib (c).

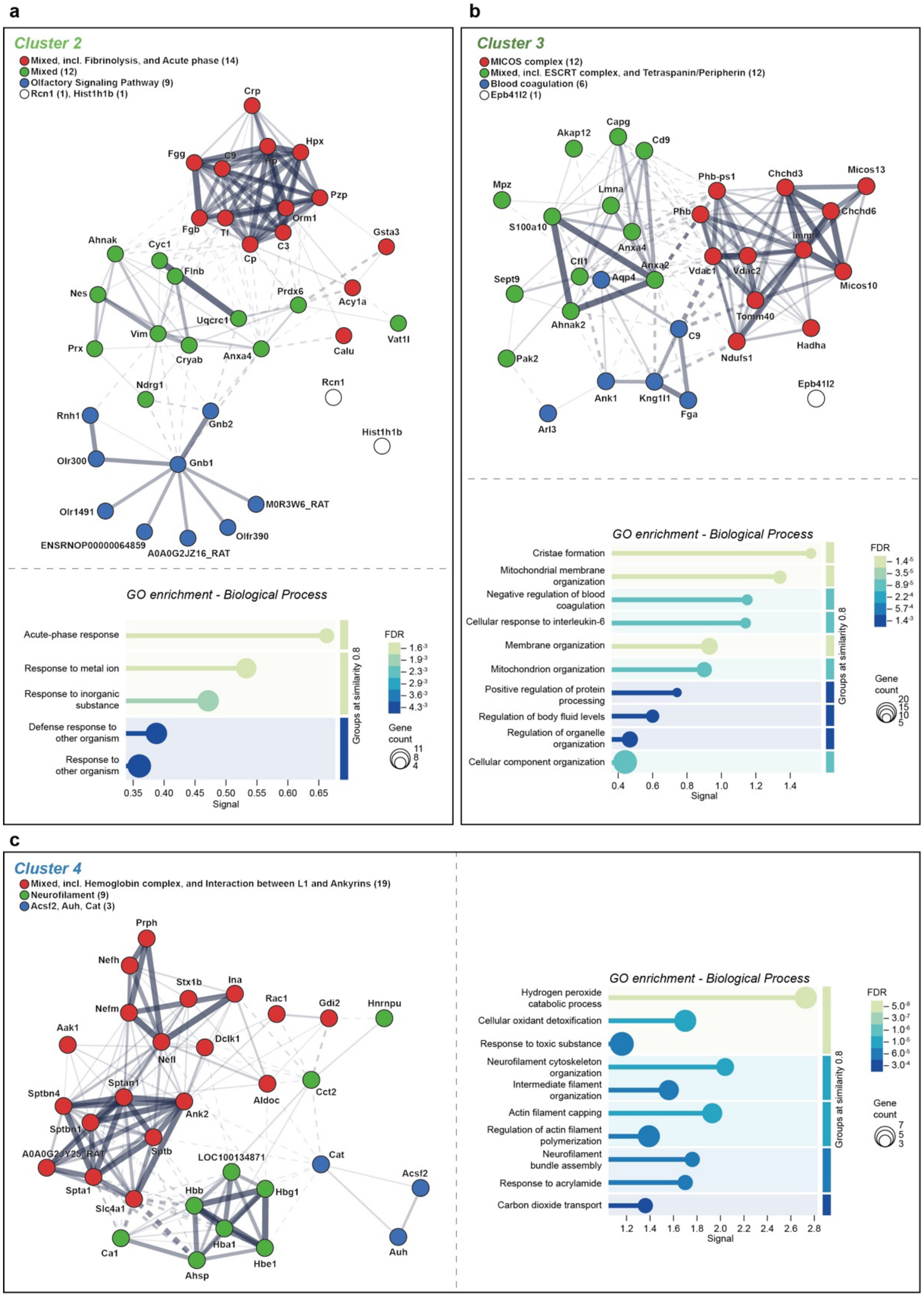

Fig. S6. Interaction networks and enrichment analysis of significantly modulated proteins in clusters 2-4. (a) Interaction network and functional enrichment gene ontology (GO) analysis of biological processes associated with significantly modulated proteins in cluster 2 (a), cluster 3 (b) and cluster 4 (c).

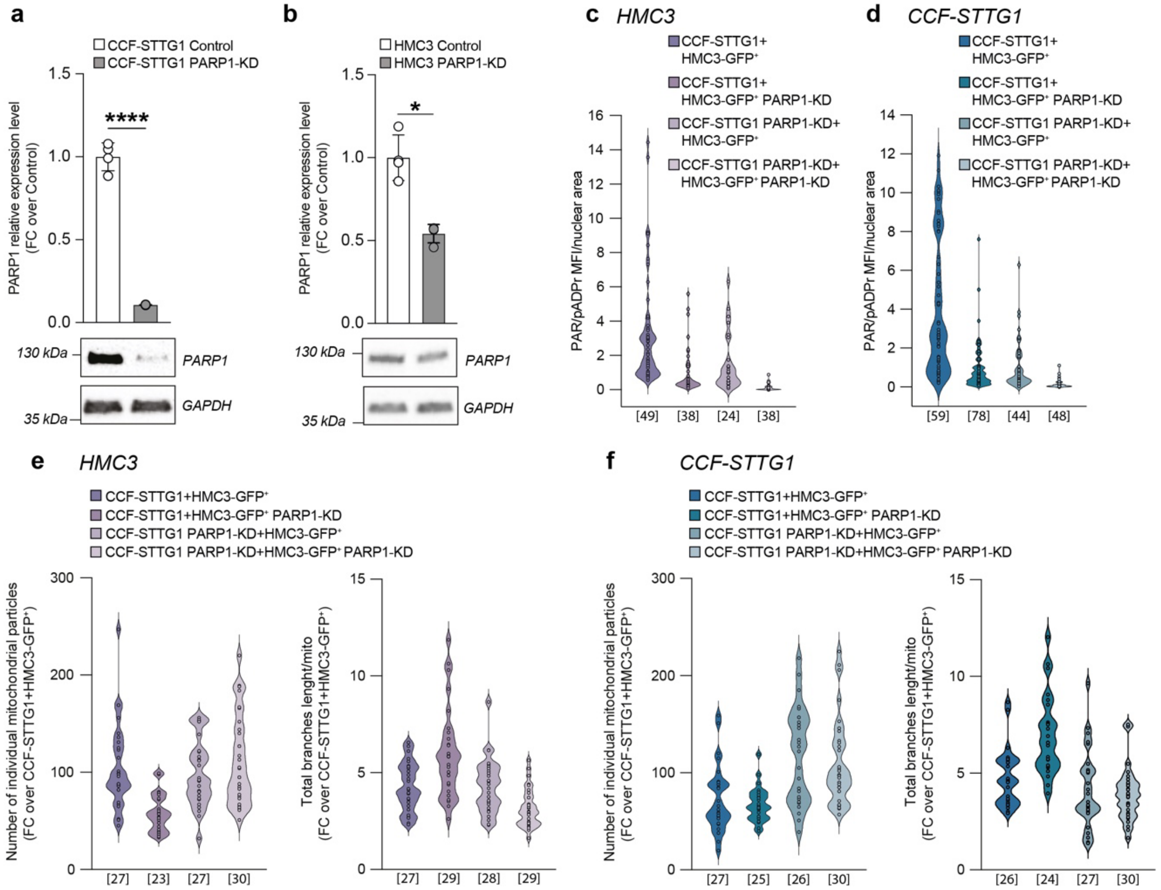

Fig. S7. PARP1 expression levels, single-cell PAR/pADPr, and mitochondrial parameters in control and PARP1-KD co-cultures. (a-b) Quantification and representative blot of PARP1 relative expression level in CCF-STTG1 control and CCF-STTG1 PARP1-KD (a) and in HMC3 control and HMC3 PARP1-KD (b). The data are reported as dot plots and means ± SDs of n = 3 biological replicates. *p-value < 0.05, ****p-value < 0.0001; one-way ANOVA followed by the Holm-Šídák multiple-comparison test. (c-d) Single-cell quantification of the MFI of PAR/pADPr over the nuclear area in HMC3-GFP^{+}/HMC3-GFP^{+} PARP1-KD cells (c) or in CCF-STTG1/CCF-STTG1 PARP1-KD cells (d) in co-cultures, related to Figure 7c-d. (e-f) Single-cell quantification of the number of individual mitochondrial particles and total branches length/mito in HMC3-GFP^{+}/HMC3-GFP^{+} PARP1-KD cells (e) or in CCF-STTG1/CCF-STTG1 PARP1-KD cells (f) in co-cultures, related to Figure 7f-i. FC: fold change, KD: knock-down.

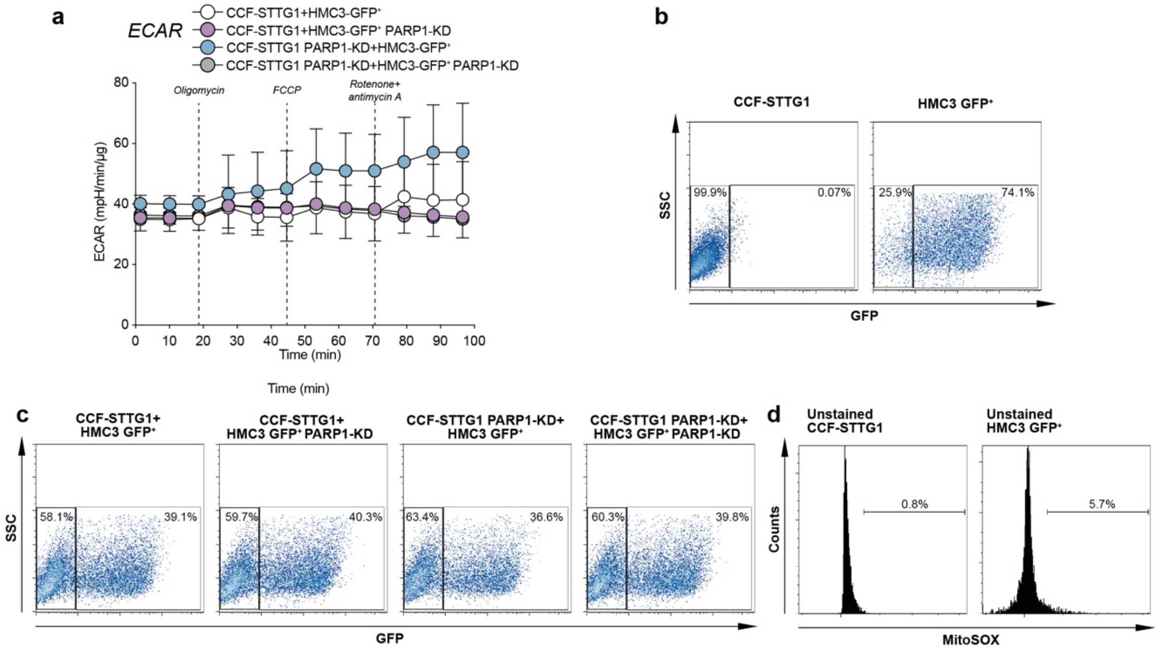

Fig. S8. ECAR and cytofluorimetric analysis of control and PARP1-KD co-cultures.

(a) Normalised ECAR of HMC3-GFP$^+$/HMC3-GFP$^+$ PARP1-KD cells and CCF-STTG1/CCF-STTG1 PARP1-KD cells in co-cultures, during sequential injections of 1.5 µM oligomycin, 1 µM FCCP, and 0.5 µM rotenone/antimycin A. Data are expressed as mean ± SEM of n ≥ 4 biological replicates. (b-c) Control cytofluorimetric analysis of GFP expression and gating in single cultures of CCF-STTG1 or HMC3-GFP$^+$ (b), and gating strategy (c) of GFP$^–$ and GFP$^+$ cells in co-cultures. (d) Gating strategy for mitoSOX in CCF-STTG1 (gated as GFP$^–$ cells) and HMC3-GFP$^+$ (gated as GFP$^+$ cells) in mitoSOX unstained co-cultures. ECAR: extracellular acidification rate, KD: knock-down.
