# Supplementary material for: PARP1 rewires neuroinflammatory and redox metabolism associated with reactive neuroglia in neuropathic pain
Source: Redox Biol. 2026 Jun 18;95:104268. doi: 10.1016/j.redox.2026.104268 (PMC13312493; doi:10.1016/j.redox.2026.104268)
Supplement: Multimedia component 2 [file mmc2.pdf]

Supplementary File Uncropped Gels and Blot

PARP1 rewires neuroinflammatory and redox metabolism associated with reactive neuroglia in neuropathic pain

Simona Denaro[1], Simona D'Aprile[2], Anna Gervasi[1], Vincenzo Russo[1], Francesco Bellia[3], Sebastiano Giallongo[2], Alessandro Lavoro[4], Saverio Candido[4], Alice Braga[5], Alexander V. Gourine[5], Giovanni Li Volti[3], Lorella Pasquinucci[6], Angela Maria Amorini[3], Carmela Parenti[7], Rosalba Parenti[1,*], Nunzio Vicario[1,*].

[1]Section of Physiology, Department of Biomedical and Biotechnological Sciences, University of Catania, 95123 Catania, Italy.

[2]Department of Medicine and Surgery, University of Enna "Kore", 94100 Enna, Italy

[3]Section of Biochemistry, Department of Biomedical and Biotechnological Sciences, University of Catania, 95123 Catania, Italy.

[4]Section of General Pathology, Department of Biomedical and Biotechnological Sciences, University of Catania, 95123 Catania, Italy.

[5]Centre for Cardiovascular and Metabolic Neuroscience, Neuroscience, Physiology and Pharmacology, University College London, WC1E 6BT London, United Kingdom.

[6]Section of Medicinal Chemistry, Department of Drug and Health Sciences, University of Catania, 95123 Catania, Italy.

[7]Section of Pharmacology and Toxicology, Department of Drug and Health Sciences, University of Catania, 95123 Catania, Italy.

***Corresponding authors:**

Rosalba Parenti (email: parenti@unict.it);

Nunzio Vicario (email: nunziovicario@unict.it).

Keywords: Reactive gliosis, central sensitization, PAR polymers, olaparib, glutathione.

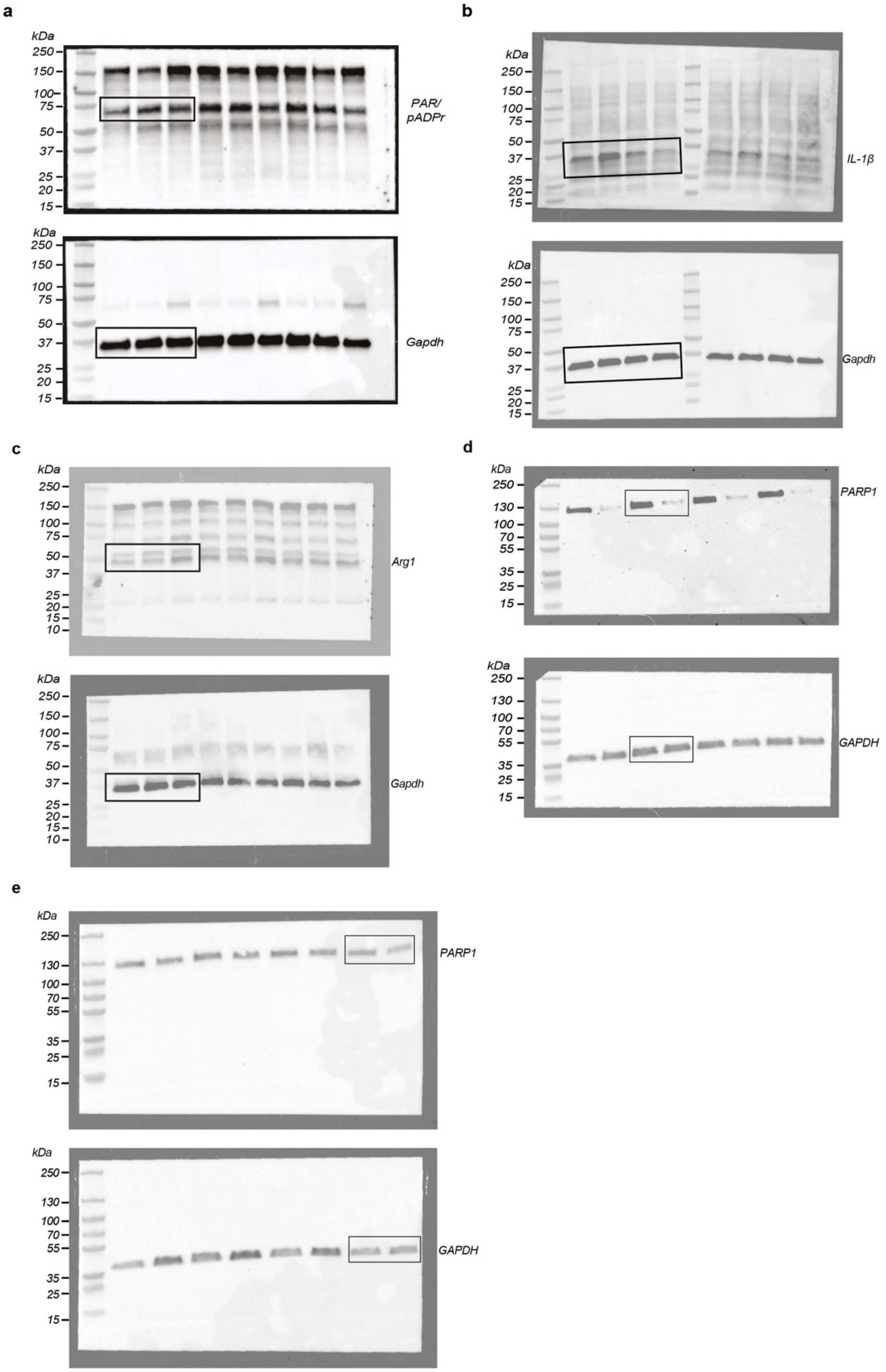

Uncropped Gels and Blots related to Figure 3, S1, S3, and S7.

(a) Uncropped membrane of the PAR/pADPr and Gapdh bands of the spinal cord protein content shown in Figure 3b.

(b) Uncropped membrane of the IL-1β and Gapdh bands of the cell protein content shown in Figure S1a.

(c) Uncropped membrane of the Arg1 and Gapdh bands of the spinal cord protein content shown in Figure S3d.

(d) Uncropped membrane of the PARP1 and GAPDH bands of the cell protein content shown in Figure S7a.

(e) Uncropped membrane of the PARP1 and GAPDH bands of the cell protein content shown in Figure S7b.
